# Development of In-Browser Simulators for Medical Education: Introduction of a Novel Software Toolchain

**DOI:** 10.2196/14160

**Published:** 2019-07-03

**Authors:** Jan Šilar, David Polák, Arnošt Mládek, Filip Ježek, Theodore W Kurtz, Stephen E DiCarlo, Jan Živný, Jiri Kofranek

**Affiliations:** 1 Institute of Pathological Physiology First Faculty of Medicine Charles University Prague Czech Republic; 2 Creative Connections s.r.o. Prague Czech Republic; 3 Department of Laboratory Medicine University of California San Francisco, CA United States; 4 Department of Physiology, College of Osteopathic Medicine Michigan State University East Lansing, MI United States

**Keywords:** education, physiology, computer simulation, modeling, Web browser, Web technologies

## Abstract

**Background:**

Simulators used in teaching are interactive applications comprising a mathematical model of the system under study and a graphical user interface (GUI) that allows the user to control the model inputs and visualize the model results in an intuitive and educational way. Well-designed simulators promote active learning, enhance problem-solving skills, and encourage collaboration and small group discussion. However, creating simulators for teaching purposes is a challenging process that requires many contributors including educators, modelers, graphic designers, and programmers. The availability of a toolchain of user-friendly software tools for building simulators can facilitate this complex task.

**Objective:**

This paper aimed to describe an open-source software toolchain termed Bodylight.js that facilitates the creation of browser-based client-side simulators for teaching purposes, which are platform independent, do not require any installation, and can work offline. The toolchain interconnects state-of-the-art modeling tools with current Web technologies and is designed to be resilient to future changes in the software ecosystem.

**Methods:**

We used several open-source Web technologies, namely, WebAssembly and JavaScript, combined with the power of the Modelica modeling language and deployed them on the internet with interactive animations built using Adobe Animate.

**Results:**

Models are implemented in the Modelica language using either OpenModelica or Dassault Systèmes Dymola and exported to a standardized Functional Mock-up Unit (FMU) to ensure future compatibility. The C code from the FMU is further compiled to WebAssembly using Emscripten. Industry-standard Adobe Animate is used to create interactive animations. A new tool called Bodylight.js Composer was developed for the toolchain that enables one to create the final simulator by composing the GUI using animations, plots, and control elements in a drag-and-drop style and binding them to the model variables. The resulting simulators are stand-alone HyperText Markup Language files including JavaScript and WebAssembly. Several simulators for physiology education were created using the Bodylight.js toolchain and have been received with general acclaim by teachers and students alike, thus validating our approach. The Nephron, Circulation, and Pressure-Volume Loop simulators are presented in this paper. Bodylight.js is licensed under General Public License 3.0 and is free for anyone to use.

**Conclusions:**

Bodylight.js enables us to effectively develop teaching simulators. Armed with this technology, we intend to focus on the development of new simulators and interactive textbooks for medical education. Bodylight.js usage is not limited to developing simulators for medical education and can facilitate the development of simulators for teaching complex topics in a variety of different fields.

## Introduction

### Background

Educators are tasked to develop innovative and creative educational materials that supplement and further enhance the traditional lecture format. This requires developing and disseminating materials that facilitate active learning, enhance problem-solving skills, and encourage discussion and interaction in small group environments. Computer simulations are one way to fulfill these requirements.

A simulation application, commonly called a simulator, comprises a mathematical model of the simulated object (a physiological system in this case) and a graphical user interface (GUI). The user interface visually represents the simulated object and its state (as computed by the model) and allows students to control the model via various inputs and controls.

Simulators are used globally to motivate students, enhance their understanding of complex topics, and foster critical thinking and problem-solving skills [1,2]. We have been creating and using simulators designed in various technologies [3] in our classrooms for many years with considerable success [4].

Complex simulators can be confusing for students and thus ineffective without additional explanation. New and effective teaching tools include interactive textbooks that integrate texts with simulators (interactive visualizations driven by models). Students can experiment with the systems and concepts under study using a simulator and thus verify and deepen their understanding. The function of the simulator is explained in the accompanying text and supplemented with suitable scenarios so that students gain maximal utility from the experience. As an example, the interactive textbooks on cardiovascular physiology by Burkhoff and Dickstein are among the first works in this field available on the internet [5] or as an iPad app [6].

Our goal was to develop a technology for the creation of similar teaching materials, but in contrast to Burkhoff [5], our innovations are designed to be platform independent and able to work offline. This technology is free and thus available for anyone to create new interactive teaching materials.

Creating books composed of texts and pictures or animations is technically not difficult. There are several suitable software tools available for this purpose. The challenging task is to include model-driven simulators that allow students to change variables, make predictions, and discover how the system works.

Production of teaching simulators is a demanding and multifaceted task requiring an interdisciplinary team of experts from multiple areas including education, graphics, modeling, and software development.

To begin the process, the educator defines the teaching objectives of the simulator, determines its main design to meet the objectives, and proposes scenarios that the simulator should be able to demonstrate. The educator also defines the demands on the model by determining the (physiological) processes that should be modeled, the parameters that are controlled by the user, and the variables that are displayed in the GUI. The educator also conceptually designs the GUI so that the system and its state is presented in a didactic way.

The modeler begins by finding a suitable model in the literature and often combines several models. If a convenient model cannot be found, the modeler derives the model from elementary (eg, physical and physiological) principles. At this point, the modeler implements the model in a programming or modeling language so that its calculation may be run on a computer. If the model is newly developed, it should be verified by comparing its results with credible reference data [7].

The artist designs the visual appearance of the GUI according to the educator’s assignment and decides the complete arrangement, including the colors and fonts. The artist also prepares all the drawings including interactive animations that may be linked to respective model variables and thus controlled by the model.

The software developer composes the GUI using the graphical components, stitches it with the model, and produces the final simulator. User controllable elements are bound with the inputs of the model. Output variables from the model are connected with the interactive graphical elements, plots, and other indicators of the GUI. The final application is then deployed on the target platform that can be a Web page or a classical native binary, and eventually, it is embedded into a broader teaching unit.

**Figure 1:**
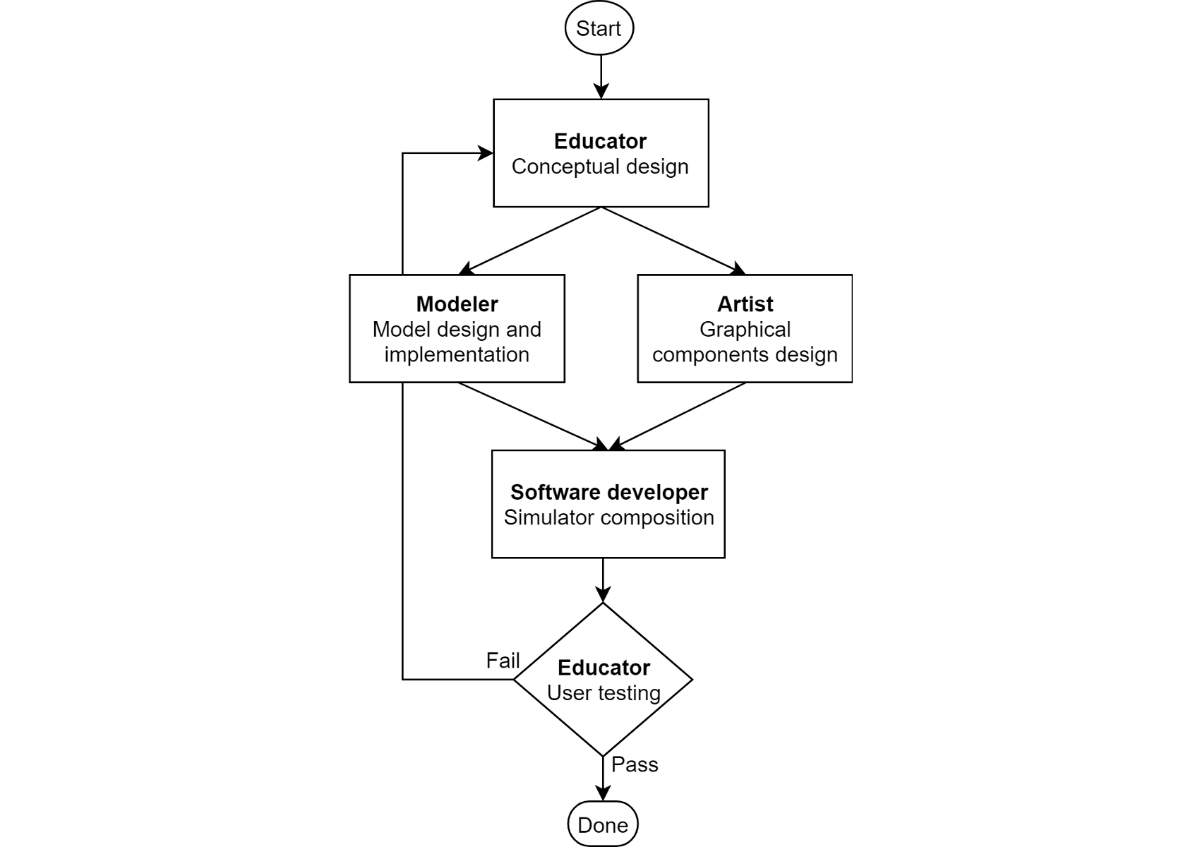
Process of simulator development.

Finally, the educator tests the simulator in an educational setting and, based on the feedback from students, may decide to iterate a new version of the simulator (Figure 1). The specific roles of the developers, development phases, and several patterns that are useful through the process are described in CoSMos methodology in more detail [8].

### Related Work and Approaches

We describe our software engineering efforts toward the development of a lightweight, easy-to-use, platform-independent, open, and standardized framework for creating interactive equation-based simulations for medical education. In this section, we discuss several existing tools related to simulator authoring to explain the reasons why we decided to develop a new toolchain and support our technological decisions.

#### Stand-Alone Executables

LINDSAY Virtual Human [9] is a 3D interactive model of human anatomy and physiology for medical education. The main focus is on anatomy. This innovative program allows *virtual dissection* and *traveling through the body* visualizations. The physiology models within LINDSAY Virtual Human are mainly implemented using the agent-based approach [10].

*LINDSAY* has been used in several projects [9]. For example, the *anARtonomy* application allows the display and manipulation of anatomical models of several human systems and organs in augmented reality. Similarly, *Zygote 3D Anatomy Atlas & Dissection Lab* is a human anatomy interactive application available for iPad, iPhone, and iPod touch. It is composed of more than 4000 anatomical structures and allows the virtual dissection of any anatomical system. Finally, *Prokaryo* is an interactive simulation application of an *Escherichia* *coli* bacterium for the Mac OS. It presents intracellular structures and uses an agent-based physiological model.

The agent-based modeling approach [11] is a useful method applied in many areas where an interaction of multiple autonomous individuals (agents) is simulated. In biology, this method is used, for example, in epidemiological modeling [12], cellular modeling (eg, immune system [13] or tumor growth [14]), or molecular modeling [15]. We focus mainly on physiological models of organs or their systems where traditional modeling based on mathematical relations is predominant. The models we base our simulators on are usually published as systems of mathematical equations, and thus, usage of the equation-based approach is straightforward. Agent-based and equation-based modeling approaches are compared by Parunak et al [16].

*Pulse Physiology Engine* [17] is a comprehensive open-source human physiology model. It is implemented including a solver in C++ and it integrates many physiological systems [18].

Teaching simulators may be based on similar comprehensive physiological models. These models are required for several applications including virtual patient simulators [19], which simulate certain diseases, complex pathological states, or the responses of the body to medications that cannot be described with a simple model.

Subsystems of complex models usually interact in complicated feedback loops, which may be difficult to understand. Therefore, simulators explaining 1 particular system or a body organ are easier to comprehend when based on a smaller model focused only on that system [20], that is, because the single system or the organ is *disconnected* from related subsystems and is not affected by the feedback loops. This method of disconnecting the subsystem is called *Ceteris paribus* (other things equal). Once the student grasps the behavior of an isolated subsystem, more complex and integrated models and simulators may be used to simulate how the subsystems mutually interact.

One important and pioneering tool in simulator production is National Instruments’ *LabVIEW*. LabVIEW is designed for control and optimization in engineering with the ability to easily connect to external hardware. Although it was not originally developed for this purpose, it can produce a complete simulator. It allows the developer to implement the model using the block-oriented approach [21] and produce the user interface.

LabVIEW produces an installable executable for multiple operating systems. It has been used for the production of physiological teaching simulators for a long period of time [22] and is currently used for that purpose [23,24], for example, simulators created by AP. Shepherd with LabVIEW are freely available on the Life Science Teaching Resource Community Web page [25]. LabVIEW was also used to control a hardware-based physiological mannequin simulator [26].

In the block-oriented modeling approach used in LabVIEW, the model comprises functional blocks (addition, multiplication, integration, and other more specific blocks). These blocks are connected by their inputs and outputs. The model input values are propagated through the block network and are modified and the output values are calculated [27]. The implementation is visual.

The disadvantage of the block-oriented modeling approach is that the modeler must know which variables are input and which are output before the model implementation. The modeler also has to derive the causality of the model evaluation, that is, the order in which the variables will be successively evaluated [27].

We find the equation-based modeling approach more convenient than the block-oriented approach. The reason for this is that with the equation-based modeling approach, the model components are implemented without any assumption about causality. The causality is resolved by the modeling tool automatically [27] when the model is being translated. Thus, the model components are reusable in different contexts. This makes the approach more convenient for complex models. We strongly prefer tools that offer the advantages of equation-based modeling. These considerations are explained in the *Modelica* section below in more detail.

The GUI is usually composed using predefined components in LabVIEW according to the tutorial [28] and the LabVIEW simulators [25]. These components are designated for the technical domain; thus, the resulting simulator usually has an industrial look.

New interactive animations may be included as a sequence of images using the *picture ring* function as recently described by Jerome [29]. Index of the image in the sequence may be bound to a model variable. This approach is convenient to control the animation with a single variable.

There are some new picture functions available [30] that could facilitate the complex animation production in recent versions of LabVIEW. The LabVIEW NXG Web Module [31,32] allows the programmer to export LabVIEW user interface to a Web browser using an approach technically similar to our own. Both these functionalities make LabVIEW possibly even more useful for teaching simulator production, but we have not found any physiological simulators using these new functions.

In addition to the block-oriented modeling issues discussed above, LabVIEW is a commercial product. This limits its use to individuals who are strongly determined to engage in modeling and simulator development because it requires an investment in a software license. In our experience, employment of commercial products may have the unfavorable effect of discouraging our occasional collaborators from participating in the simulator development.

The iPad version of cardiovascular textbooks by Leisman and Burkhoff [6] is another example of a standalone simulator.

In general, the distribution of a standalone executable may bring installation issues and discourage its use. The executables are platform dependent and must be generated or compiled for each platform separately.

#### Browser-Based Client-Server

Examples of client-server simulators include JustPhysiology [33] and the Web version of the cardiovascular textbook series by Burkhoff et al [5]. Several technical approaches of how to realize client-server Web-based simulators with special emphasis on Modelica modeling are discussed by Meyer et al [34].

A platform for interactive Modelica content called Modelica University was developed by Tiller and Winkler [35]. Simulators created using this platform are included in the *Modelica by Example* Web-based book by Tiller [36]. The simulator GUI is composed manually in JavaScript.

Žáková and Cech [37] implemented a Web service that runs a Modelica model and implements the JSON-RPC (JavaScript object notation remote procedure call) protocol [38] for the communication with a client application. This application allows individuals to remotely upload, translate, and simulate a model. There are no special tools for the client application production. The client applications using this server back-end may be created using any suitable technology. Several teaching simulators using this technology were created [37], mostly for a course of Control Engineering. We did not find a website of the project nor any realized simulators; therefore, we assume that the project is available for the author’s purposes only.

Simulators using the browser-based client-server approach are already platform independent and do not require any installation. Unfortunately, usage in a classroom or during a lecture, where a multitude of students may use the simulator simultaneously, ramps up the computational and bandwidth demands on the server. This can be a major disadvantage.

Latency can be an additional disadvantage of this approach. In fast-paced games, latency greater than approximately 50 ms starts degrading the user experience [39]. Although it is clear that teaching simulators are not as sensitive to latency as real-time gaming, low latency contributes to optimal user experience. For example, when the interaction with the UI produces instant effects, users can continuously move a slider to control a model parameter and quickly observe progressive responses of the system.

The total latency is caused by model evaluation, graphics rendering, and, in the case of client-server architecture, the network communication delay. The internet connection must be fast, and the server must be close enough to achieve low network latency [40].

To solve the issue with an unstable connection in a client-server architecture that would cause the animation to stutter, we would use a caching strategy such as has been done by Brukhoff [5], which would enable us to produce smoothly running animations.

Another disadvantage of this approach is that these simulators require continual connection to the server back-end, and thus, this approach is not suitable for interactive textbooks that need to work offline.

We consider the client-side approach more suitable because renting a cloud service providing enough computing power to serve numerous end users simultaneously and having data centers distributed around the globe to achieve a reasonable round-trip time may become expensive for many individuals and small teams.

On the other hand, the client-server approach is convenient in situations when the client device has insufficient performance to evaluate the model in reasonable time, and the server may deliver the results faster.

#### Browser-Based Client Side

To counter the drawbacks of stand-alone and client-server solutions, simulators could be run directly on the client, that is, the browser. Client-side simulators are platform independent. Additional advantages include that they do not require any installation, do not put a heavy load on the server, and can operate on a slower internet connection or fully offline. A major difficulty with this approach is the need to rework any existing platform into a JavaScript codebase. Some pioneering work has already been completed by Wagner [41].

Specifically, we developed a Bodylight(.Net) simulator framework [42], built on a Microsoft Silverlight browser plug-in. In this project, the OpenModelica compiler was extended so that it could translate the model in *C#* language. The OpenModelica runtime and solvers were rewritten manually into the *F#* language. A problem with this approach was that OpenModelica is developing rapidly, and the rewritten runtime must be updated constantly to stay compatible with the rest of the system. The Silverlight plug-in was finally discontinued, and the framework became obsolete and useless.

The *openmodelica-javascript* project by Tom Short [43] extends OpenModelica so that it can compile the models and simulation runtime automatically to JavaScript using *Emscripten* (we also use emscripten). Browser-based simulators comprising text boxes to input parameter values and plots to visualize results can be created easily. As the simulation runtime code is generated automatically, it is much less laborious to keep the system compatible with OpenModelica compared with our obsolete Bodylight(.NET) solution based on Silverlight. The disadvantage is that this project relies only on OpenModelica, and other modeling tools are not supported. Furthermore, more complex GUI elements including sliders and interactive animations are not supported (although it is probably possible to implement them manually). Another concern is that it does not offer any tool to easily compose the GUI, for example, in a drag-and-drop fashion. Owing to the changes in OpenModelica, the openmodelica-javascript project does not currently work with recent versions of OpenModelica (personal communication with Tom Short).

#### Related Software Frameworks and Libraries

In addition to the technologies and the software libraries described in the Methods section, there are several other technologies that could possibly be beneficial for use. Here, we discuss some of them.

*BabylonJS* [44] is a free JavaScript 3D engine for games and other 3D visualizations in a Web browser using WebGL. It has several applications for medical e-learning (electronic learning). For example, EducaAnatomia3D is a serious game for human anatomy education [45]. The 3D models may be created in Blender [46] (a free 3D modeling tool) and exported for use in BabylonJS [47]. This combination of BabylonJS and Blender could conveniently extend our toolchain that aims to be based on free tools.

*Tree.js* [48] is another JavaScript engine for 3D visualizations in a Web browser based on WebGL, with applications in medical e-learning [49]. If we decide to include support for 3D graphics, for example, to enable more accurate anatomical visualizations, we could use one of these 2 engines.

Another important game engine and development platform is *Unity* [50], developed by the Unity Technologies company. It allows the innovator to create both 2D and 3D interactive experiences. Unity is available in several versions, and one of the versions is free. Another important aspect of Unity is that it has its own development editor. The main programming language is C#. Unity allows the innovator to build applications for many different platforms. One of the target platforms is WebAssembly [51], which allows the produced simulator to be run in the browser. It is used for the development of serious e-learning games [52-56] in many fields including medical education [57]. We have previously used Unity in combination with Bodylight(.Net) framework in *Surviving Sepsis - a 3D integrative educational simulator* proof-of-concept project (for the Windows Store platform) [58]. Unity is highly convenient to develop complex serious games. If we decide to focus on serious game development, we would consider using Unity, although it is a commercial product.

### Goals for the New Toolchain

There are excellent Web technologies available based on JavaScript, including many useful frameworks and libraries that almost equalize the capabilities of browser and native applications. There are also great modeling tools available. The problem is that the models are deployed in native programming languages (C or C++) so that they cannot be run in a browser. Our goal was to fill this gap and allow the use of the dedicated modeling tools and enable running the resulting models in the browser without a server-side back-end. On the basis of our previous experience, we have formulated the following requirements on the tool for simulator production:

- *Browser based:* to achieve widespread compatibility and avoid the necessity of an installation process, which could discourage many users.
- *Client side:* so that a multitude of users are able to run the simulator simultaneously without renting expensive computing hardware from a third-party provider.
- *Future proof:* the toolchain should be based on standards, which are unlikely to become obsolete in a reasonable time frame.
- *User friendly:* to enable all those participating in the simulator production to use an appropriate tool for their task so that the work is efficient and pleasing and the resulting simulator is satisfactory.
- *Equation based:* to facilitate model development, their implementation in an equation-based language, preferably Modelica (advantages discussed in the *Methods* section) should be allowed.
- *Open source* and *freely available:* our aim in building the toolchain is to use relevant open source projects and reap the fruits of the hard work of the developers and also contribute back and make the toolkit available for anyone to use under a copyleft license. Open-source licensing should also allow the project to grow in the future and nurture the collaboration between the developers and users. It is especially important for the modeling tool to be freely available. We often collaborate on model development with colleagues from different workplaces, and with a freely available tool, anyone can participate.

There are many tools available, which are useful in the process of the simulator production, and they meet several of the requirements. However, we were unable to find a single tool or toolchain meeting all our requirements. To address this limitation, we developed a toolchain that meets all our specific requirements.

## Methods

We created a new toolchain within the confines of the specified requirements, using the following technologies.

### Modelica

Modelica [59-61] is an equation-based, object-oriented, open standard language for the simulation of complex physical systems.

In the equation-based (acausal) approach, the model is composed of equations that state the relationship between the variables (eg, *x*^*2*^ *+ y*^*2*^ *= 1*) as opposed to assignments where 1 output variable (on the left-hand side) is assigned a value of an evaluated expression (right-hand side; eg, *y:=* sqrt *(1 – x*^*2*^*)*) [62]. The causality of the evaluation (order in which equations are evaluated and which variables are calculated from which equation) does not have to be known in the modeling phase. It is derived automatically by the solution tool later [62]. We prefer this approach over block-oriented modeling because, in equation-oriented modeling, the modeler saves the labor of the causality derivation. Moreover, because the model components are implemented without any assumption about causality, they may be easily extended or later reused in various models, where the causality differs [62]. This is very useful in creating reusable component libraries. We base our simulators on models that are usually published as equation systems, and thus, their implementation in the equation-based approach is straightforward.

**Figure 2:**
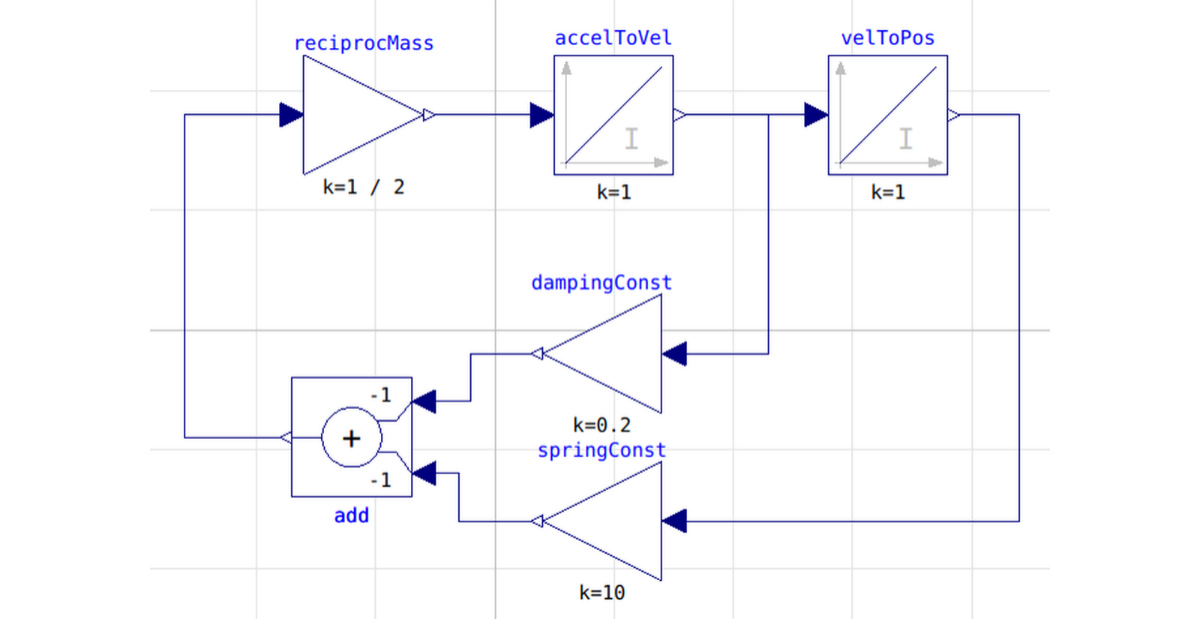
Harmonic oscillator model implemented using block-oriented approach in Modelica.

**Figure 3:**
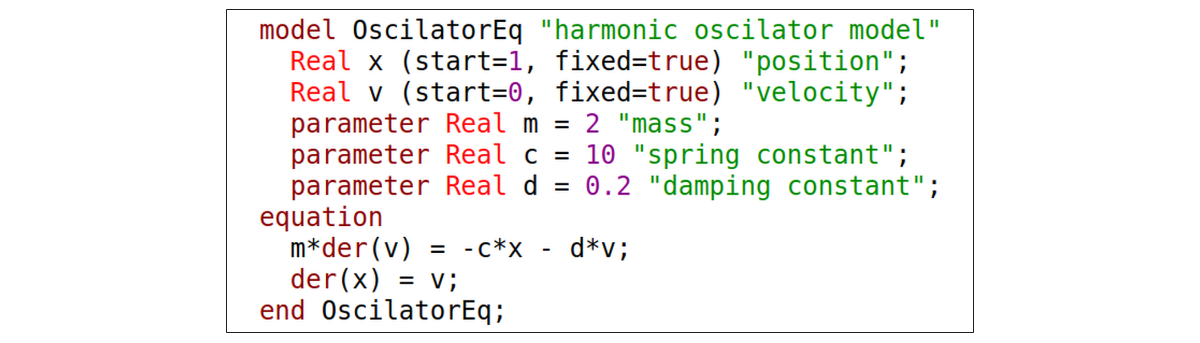
Harmonic oscillator model implemented using equation-based approach in Modelica.

Below, we illustrate the difference between both approaches with example of harmonic oscillator model described in the equation *m∙x'' = –k∙x – c∙x'*, where *x* is position, *m* represents mass, *k* represents spring constant, and *c* represents damping constant. Implementation of this model using the block-oriented approach in Modelica (as Modelica supports the block-oriented modeling as well) is illustrated in Figure 2. The code of the same model implemented using the equation-based approach in Modelica is listed in Figure 3. The equation-based (acausal) and block-oriented approaches are compared in several reports [27,62,63].

**Figure 4:**
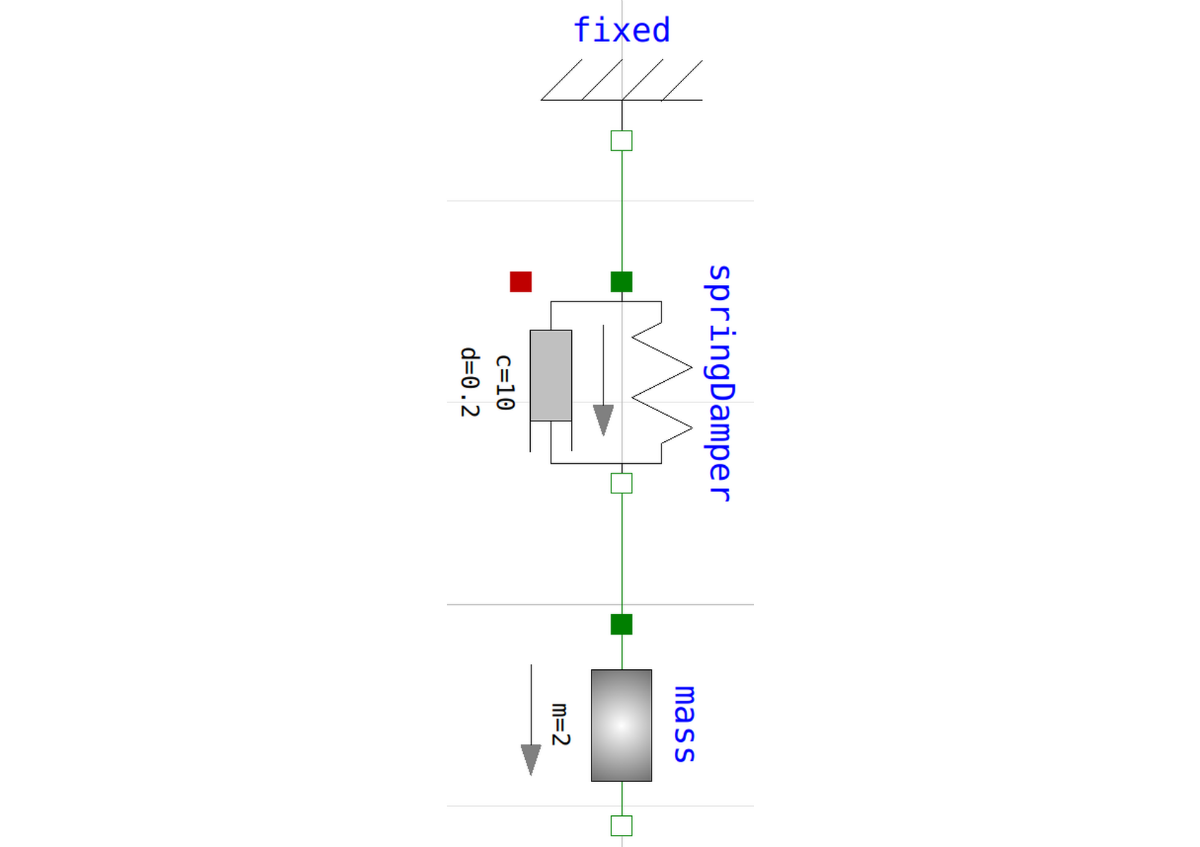
Harmonic oscillator model implemented using Modelica Standard Library.

Owing to its *object-oriented* nature, Modelica scales very well for large complex models [62]. Basic model components (classes) are defined by equations. Components have connectors. More complex components are composed of other components that are connected using their connectors in a visual way. These connections are rendered into additional equations binding the variables of the connected component. Inheritance is also supported and enables significant code reuse. Numerous Modelica libraries of predefined components from many different domains exist. A Modelica library for physiology called Physiolibrary was developed in our laboratory [64,65]. The harmonic oscillator model implemented using Modelica Standard Library is illustrated in Figure 4. The model appearance intuitively explains the modeled system.

Modelica is supported by many different modeling tools, primarily because of the fact that it is an *open standard*. This is important because it allows the use of multiple tools that broaden and expand the modeling capabilities. We use either OpenModelica [66], which is free, or Dassault Systèmes Dymola [67], which is a more advanced, but a proprietary modeling tool.

Modelica is an open-standard language supported by open-source tools and, as such, is ideal for model sharing [68].

### Functional Mock-Up Interface

Functional Mock-up Interface (FMI) [69] is a standard for exchanging and cosimulation of dynamic models between different independent tools and applications. Both Dymola and OpenModelica allow for the model to be exported as a Functional Mock-up Unit (FMU). When exported in the mode *FMI for Co-Simulation*, the unit contains a simulation runtime, which takes care of the model calculation and execution. The data exchange with the outside world is restricted to discrete communication points, and between them, the unit is solved independently by the included solver [70].

The FMU can be exported with source code necessary to compile binaries, which implement the Co-Simulation standard. This ability is very advantageous for our purposes as we can compile the source code into a Web language and have full access to the FMI for Co-Simulation features in the browser, which allows us to interact with the model easily.

The FMI standard ensures compatibility with future versions of both OpenModelica and Dymola. Furthermore, the Bodylight.js system can be easily adapted to support other simulation tools implementing the FMI export option.

### JavaScript

JavaScript is a multiplatform, object-oriented, interpreted programming language [71]. It was originally developed by Brendan Eich in 1995. JavaScript is the most notable implementation of the ECMAScript standard. It is supported by all recent important Web browsers [72]. It is widely used today to enable interaction and dynamic behavior on websites.

### JavaScript Libraries

We use the *GrapesJS* open-source Web Builder framework [73] as our HyperText Markup Language (HTML) layout engine. GrapesJS allows us to use the drag-and-drop approach for designing how the simulators appear. There is a set of built-in blocks available to build the app, and it allows for easy customization and production of additional blocks as well. Available configuration panels enable the programmer to edit properties and the behavior of the components on the canvas. The modular design of GrapesJS allows us to hook into its user interface and extend it as a base of the main user experience.

*EaselJS* [74] is a component of the CreateJS toolkit. It allows for easy manipulation of HTML5 Canvas elements and can be used to create games, art, and other graphical experiences. More importantly, for our interests, Adobe Animate natively supports the export of animations to EaselJS. We can either use Adobe Animate to design the animations or EaselJS directly to create original animations.

Finally, *Plotly.js* [75] is a high-level, declarative charting library implemented in JavaScript. It can be used to display many types of charts and graphs.

### WebAssembly

WebAssembly [76] defines a binary instruction format to be executed inside a stack-based virtual machine. Its primary use is to be implemented inside Web browsers, aiming to provide code execution at near-native speeds. The binary format is designed to be efficient with respect to size and load time, reducing the time necessary to transmit and load the code.

WebAssembly is a target of compilation for high-level programming languages such as C or C++, enabling the compilation of existing and new code written in C/C++ to the browser platform. The compiler that facilitates this is the open-source project, Emscripten [77,78], which we use to compile source code inside the FMU. The compiled code can be considered obfuscated to the level similar to those with other binary instruction format representations. The algorithms can be disassembled into a pseudocode and with great effort and investment of time can even be reverse engineered [79]. For most practical purposes, the models can be considered obfuscated when compiled to WebAssembly.

## Results

### Overview

The new Bodylight.js toolchain uses the work of several third-party open-source tools and compilers and other newly written tools. The Web page [80] of this project includes documentation and tutorials. In this section, we describe the complete workflow in more detail and define all the processes involved and the tools used. We focus particular attention on the newly developed *Bodylight.js Composer*, which enables one to create the final simulator by composing the GUI using animations, plots, and control elements in a drag-and-drop style and bind them to the model variables.

### Model Processing

**Figure 5:**
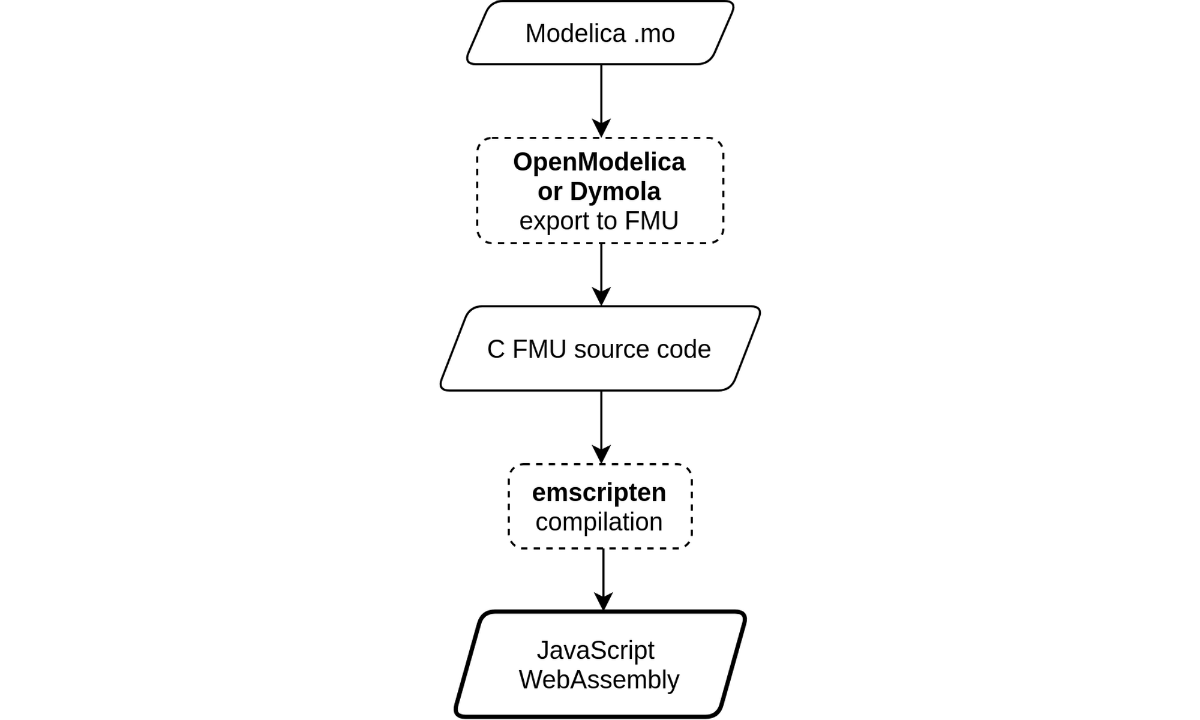
Model workflow—Modelica model is exported from OpenModelica or Dymola to a Functional Mock-up Unit (FMU), which is compiled using emscripten into WebAssembly and JavaScript.

The model workflow is schematically illustrated in Figure 5. The model is written in Modelica, usually inside one of the popular Modelica IDEs such as the open-source OpenModelica or the proprietary and well-established Dymola.

The next step is to export the model into an FMU. Dymola supports the export of source code inside the FMU under their *Source code generation* license [81], and OpenModelica seems to always export the source code inside the FMU. Unfortunately, OpenModelica, as of the date of publication, only allows the export of a Euler solver, which is insufficient for many models.

The FMU then needs to be compiled into WebAssembly and JavaScript. To complete this step, we have prepared a Docker container, which uses Emscripten to automatically compile FMUs into Composer compatible files [82].

The container accepts FMUs generated by Dymola with the *source code generation* license and FMUs generated from OpenModelica on a Linux platform. The requirement for Linux is because of FMUs from OpenModelica being generated with makefiles, which are not only platform dependent but also machine dependent. To facilitate easier environment setup for the OpenModelica part of this workflow, we have prepared another Docker container, which uses OpenModelica to export FMUs automatically [83].

### Animation Processing

We use Adobe Animate [84] in the process of designing the interactive animations. Adobe Animate supports native export to HTML5 and JavaScript using the library EaselJS. This process is fairly painless as all the post processing of the exported JavaScript code is handled by the composer. Furthermore, the user can opt to write all EaselJS directly without the need to use the Adobe product.

### Bodylight.js Composer

*Bodylight.js Composer* is the main development focus of this project. Composer is a single-page application that can easily bring together models, animations, and control elements.

The core of Composer is built on React, an established and very popular JavaScript library. The HTML layout engine is provided by GrapesJS, around which the rest of the application was shaped. Composer allows the user to easily design an interactive HTML simulator. There are also several input and output widgets available. The *range* widget handles the control of the model variables. The *chart* widget uses plotly.js to display an output from the model in interactive charts. Toggle widgets and buttons can control Boolean values, and labels are used to display values.

The Composer is equipped with *actions* that are user-generated snippets of JavaScript code and can be attached to events of other widgets. For example, one of the prefilled actions is *reset model* with a model as a parameter. Users can attach *actions* to an *onclick* event of the *button* widget and select the appropriate model to reset. The *animate* widget is used to import complex animations created in Adobe Animate. These can contain continuously playing animations, whose speed and direction can be controlled by any model variable. Furthermore, it can control positional animations, where the timeline position is directly controlled by a model variable.

**Figure 6:**
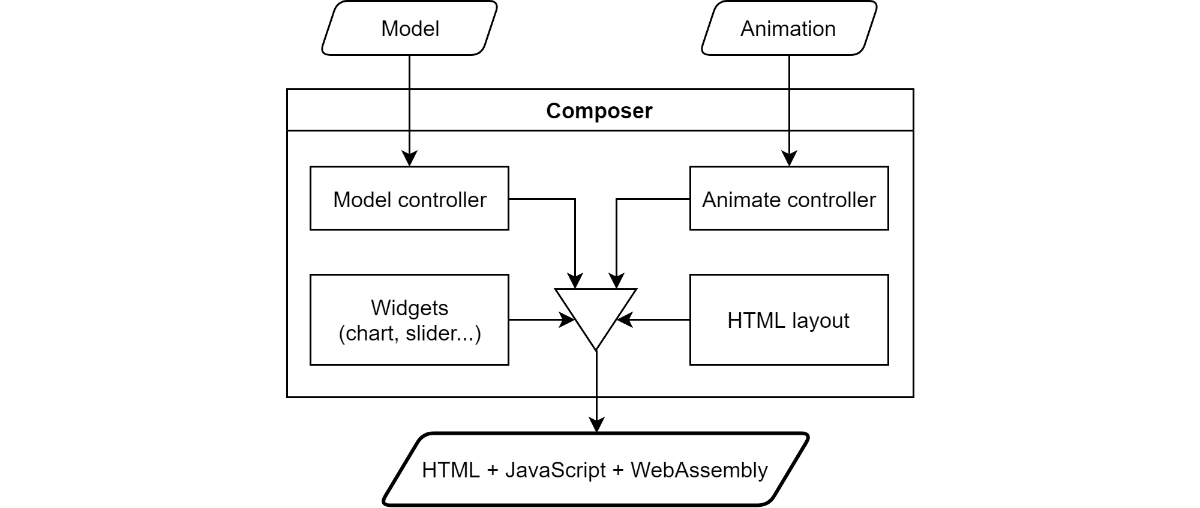
Composer workflow scheme—HTML (Hypertext Markup Language) layout is created, animations are loaded, the model is loaded, model and animation variables are bound, and control elements and plots are added and bound with model variables.

**Figure 7:**
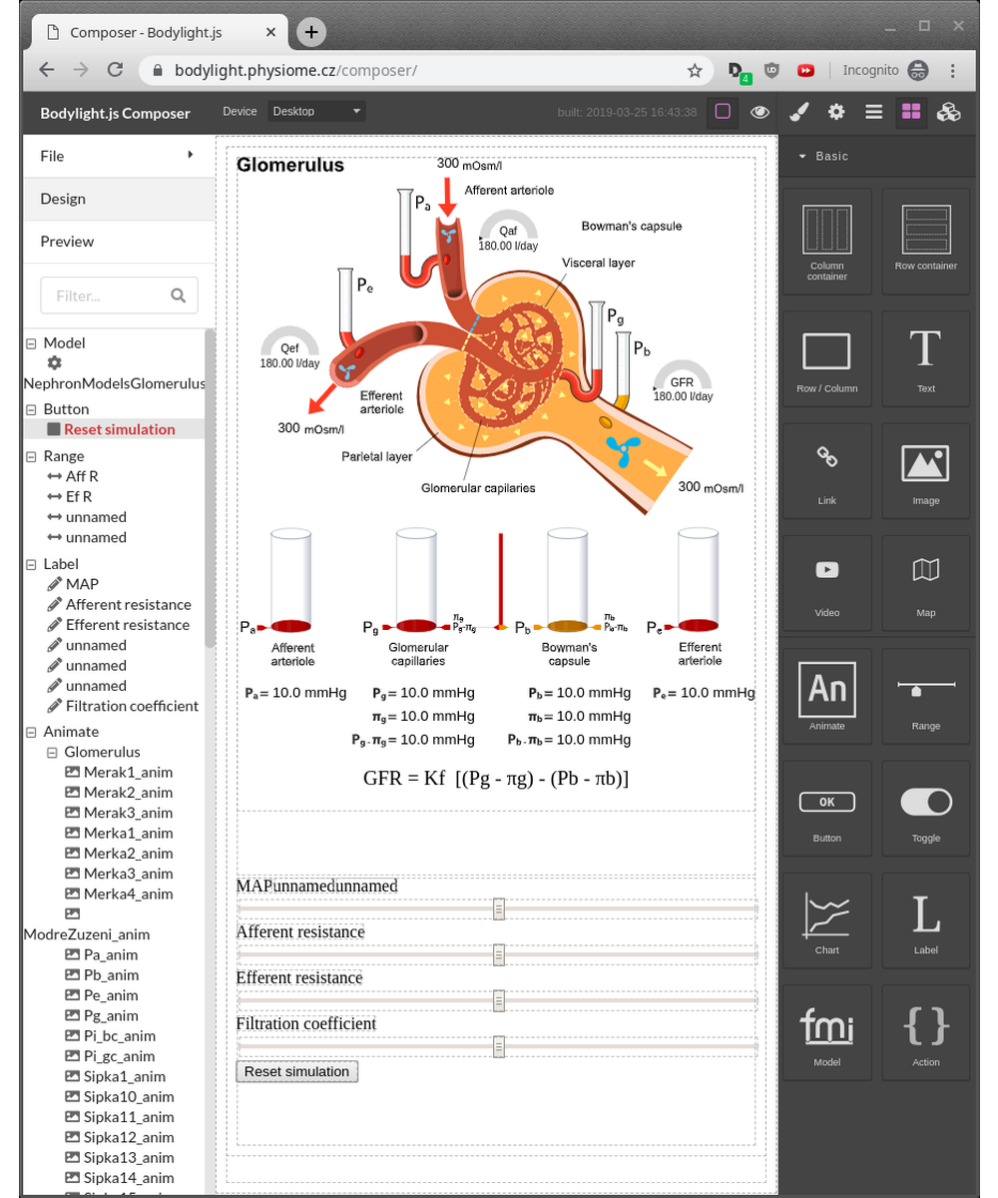
The Glomerulus application page inside Bodylight.js Composer.

Users can also save and open shareable project files. The final export from the Composer is a stand-alone HTML file, containing the JavaScript and WebAssembly code. Composer workflow is depicted in Figure 6 and the composer itself in Figure 7.

**Figure 8:**
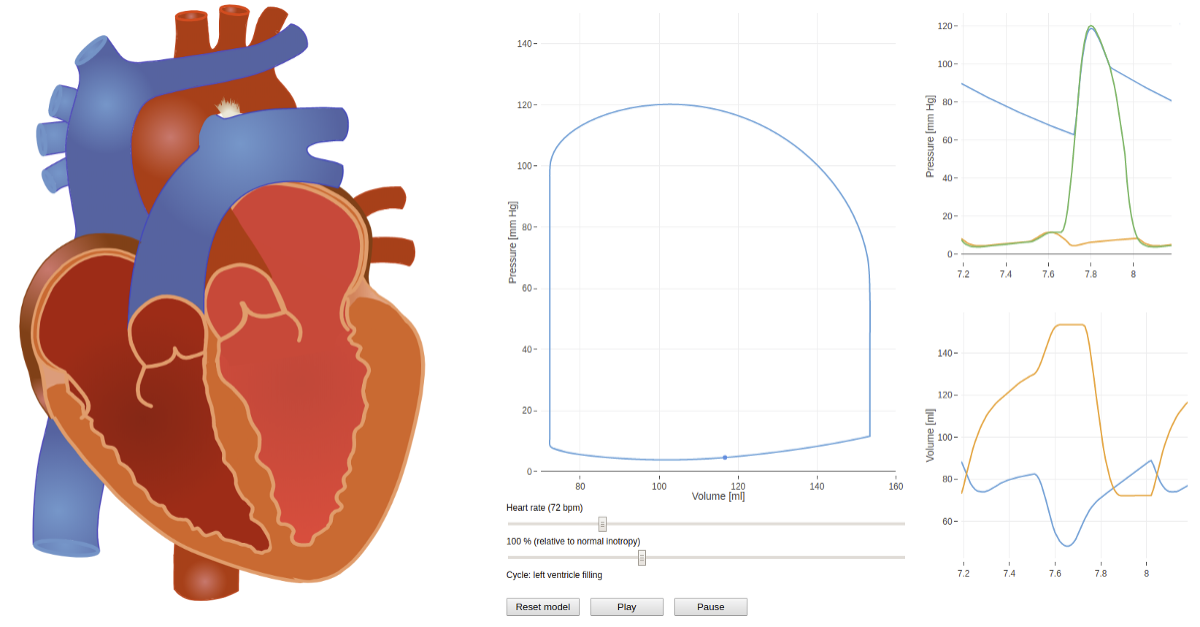
Pressure-volume loop simulator is a result of the more advanced tutorial.

We recommend readers to view the video tutorials on the Bodylight.js Web page [80] to get a better understanding of how Bodylight.js Composer works. The simple *Bouncing Ball* video on the main page demonstrates how to build a simulator comprised a simple interactive animation, a plot, a slider, and a reset button. It is also included in the Multimedia Appendix 1. There are 2 additional video tutorials available in the *Documentation* section of the Web page. The *Simple Project* tutorial [85] demonstrates how to build a simulator composed of sliders, plots, and a reset button. It is logically sectioned into different episodes. The steps are also explained below the tutorial videos. The second tutorial available is the more elaborate *Physiological Application* tutorial [86]. This tutorial demonstrates several advanced features for building a real simulation application of pressure-volume cardiac loops depicted in Figure 8. The additional features explained include creating a parametric plot, adding a start-stop button, controlling model parameters by events, and applying user functions on model variables. For example, descriptions on how to round the values or display string messages and other features are discussed. The translated models and the Composer project files are included in both tutorials.

### Original Simulators

Several simulators were created using Bodylight.js. In this section, one of the simulators is presented to demonstrate the capabilities of Bodylight.js. First, some basic physiology is introduced, and then the simulator is described, and the features of Bodylight.js are highlighted.

#### Nephron Simulator

The main purpose of the kidney is to produce urine and control its composition. The functional unit of the kidney is the nephron. There are approximately 2 million nephrons in a pair of kidneys. The nephron is composed of the glomerulus and a system of tubules. The glomerulus is a network of capillaries. The blood is filtered across the capillary walls; thus, primary urine (filtrate) is produced (approximately 180 L/day). The filtrate then flows through the system of consequent tubules, each having a slightly different function, where the water and specific solutes are reabsorbed so that the appropriate amount of urine with the required composition to maintain homeostasis is produced and excreted.

These processes are explained visually by the simulator. For simplicity, the stimulator only focuses on water and sodium. The simulator is available online [87] and is attached as the Multimedia Appendix 2. Multimedia Appendix 2 also includes the additional required libraries for offline use. A more detailed description of the simulator including its didactic objectives, models, and implementation is beyond the scope of this paper. More information is available in our recent work [88].

##### Glomerulus Screen

**Figure 9:**
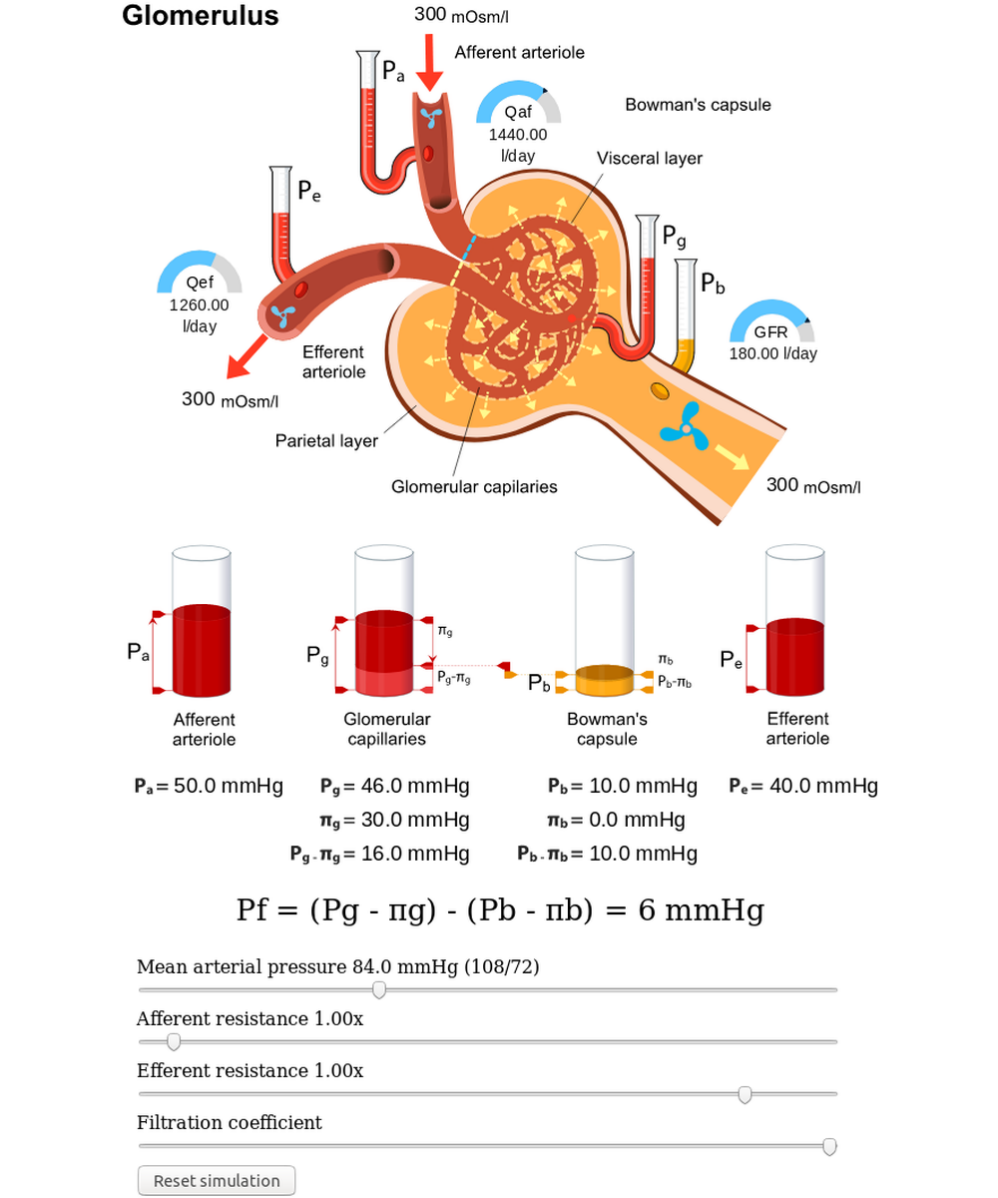
Glomerulus. Pressure is visualized by the liquid-column indicators and flows by the propellers and half-circle indicators. The red arrows symbolize the blood flow direction, and yellow arrows represent urine flow direction. GFR: glomerular filtration rate.

Figure 9 depicts the glomerulus screen. Resistances of the arterioles, mean arterial pressure, and the filtration coefficient are controlled with the sliders. Changes of these parameters affect pressures and flows in the system. Pressures are depicted by the liquid-column gauge and flows through the tubules by the speed of the propellers and the half-circle indicators (normal values are marked by a tick). Flow through the vessel walls is shown with the width of the dashed moving arrows. The hydrostatic and oncotic pressures are also indicated by the cylinders below the glomerulus. All the indicators are interactive and react to changes in values of the model variables.

Note that the numeric values of the flow indicators and the pressure values in the formulae are also controlled by the model. There is also a *Reset simulation* button which sets all the parameters to default values.

There is no time evolution in the model, and everything is computed in the initialization phase. The simulator operates in the so-called *One shot* regime. This means every time parameters are changed by the sliders, the model is automatically recalculated and the GUI is updated accordingly. The same also holds for the next screen.

##### Complete Nephron Screen

**Figure 10:**
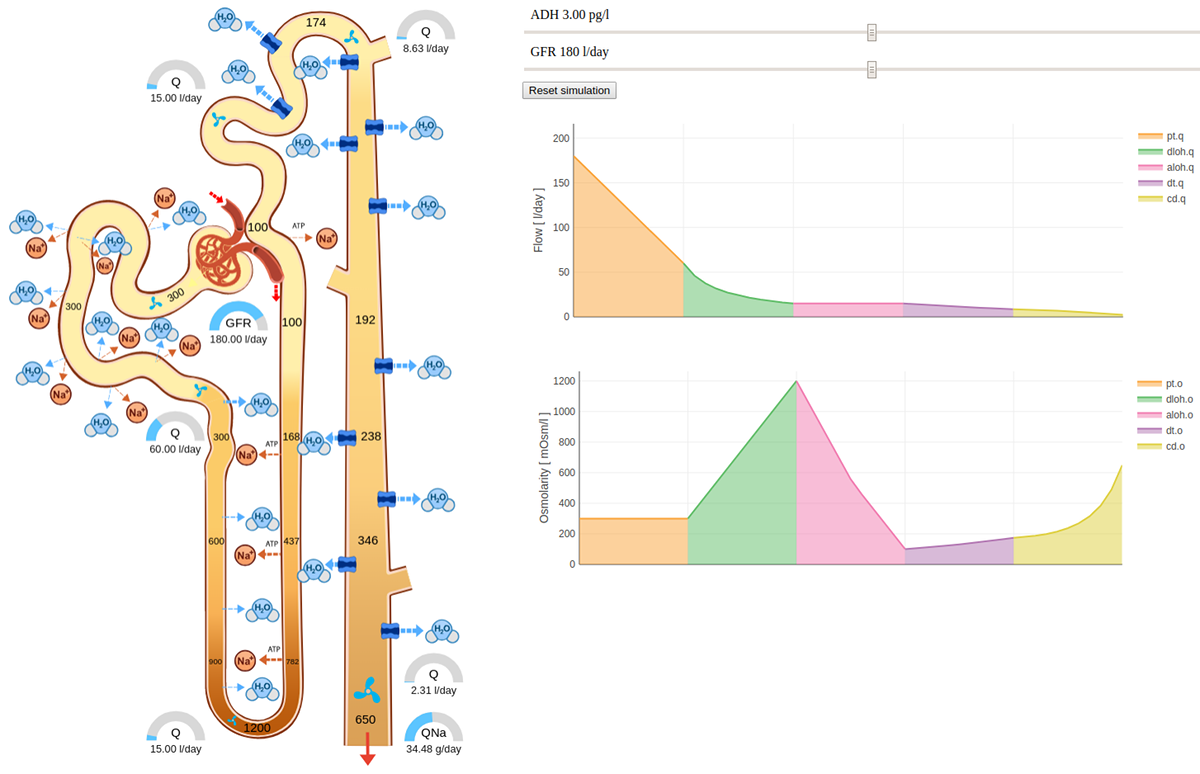
Complete nephron. Half-circle gauges show urine flow rate, the lightness gradient inside the tubules visualizes osmolarity, dashed blue and orange moving arrows visualize water and sodium flow through the tubule walls and the blue channels indicate tubule water permeability. Flow and osmolarity are plotted in the charts (individual sections in a different color). ADH: antidiuretic hormone; GFR: glomerular filtration rate.

The simulator of the complete nephron is shown in Figure 10. The glomerular filtration rate and the antidiuretic hormone (ADH) parameters are controlled with the sliders. Filtrate flow rate and the sodium mass flow rate are visualized by the half-circle indicators. Osmolarity (proportional to solute molar concentration) of the filtrate is determined by the numbers and the lightness gradient inside the tubules. Water and sodium flow through the tubule walls are determined by the width of the blue- and orange-dashed moving arrows. The amount of ADH and the consequent tubule water permeability is visualized by the varying width of the blue water channels in the last section of the tubule.

Flow and osmolarity are functions of distance along the nephron tubules and are plotted on the chart. The individual sections, whose variables are discretized using an array in the model, are highlighted in different colors for easier identification. The legend, the labels, the line color, and the width as well as several other plot properties are also adjusted.

#### Blood Circulation

**Figure 11:**
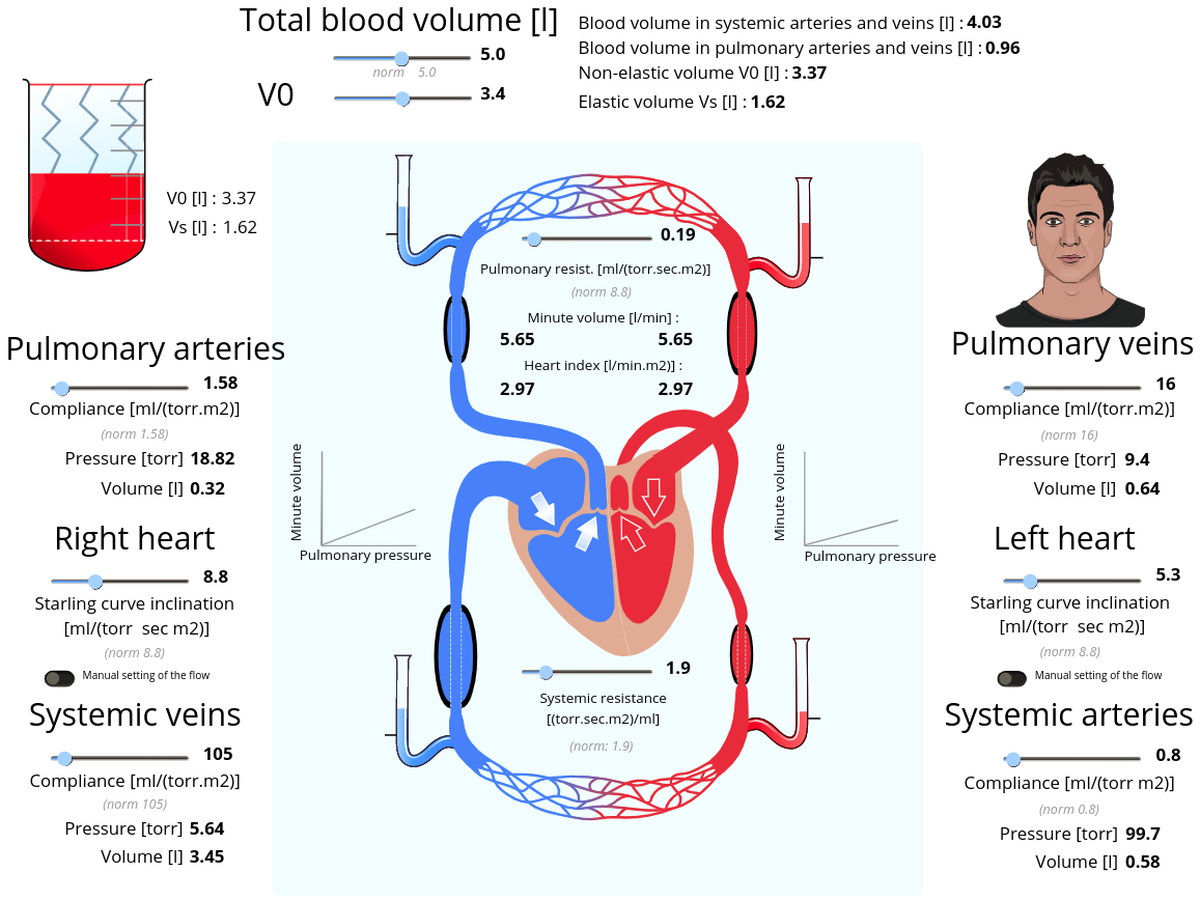
Blood circulation simulator. Pressures are visualized by liquid-column gauges, blood volumes are depicted by the width of the sections of blood vessels with black borders, blood flow rates are visualized by the frequency of blinking arrows. Changes in arterial pressure are also reflected by animated changes in facial expression and color.

The blood circulation simulator (Figure 11), available online [89], is another example illustrating the use of Bodylight.js Composer. The simulator depicts 2 general blood vessel circuits: (1) a large systemic circuit distributing blood to the body and (2) a small pulmonary circuit running through the lungs. The circuits are connected through the heart. The simulator is based on a basic physical model with the default parameter values fitted to clinical values to provide meaningful results. Students can modify various model parameters: (1) the compliance of systemic and pulmonary arteries and veins (reciprocal to elasticity), (2) the slope of the Starling curve reflecting cardiac contractility and cardiac output, (3) the total and nonelastic (unstressed, V0) blood volume, and (4) the pulmonary and systemic resistances. The simulator is supplemented with animated figures; therefore, the system response on external perturbation can be followed graphically (intuitively) as well as through the numeric values calculated by the model running in the background. Note that the human face animation is also controlled by arterial pressure, with facial changes in expression and color representing low, normal, or high pressures.

#### Pressure-Volume Loop Simulator

A cardiovascular simulator (Figure 8), available on the Web page [90], displays the pressure-volume loops of the left ventricle. It is based on our Modelica implementation [91] of the model by Burkhoff and Tyberg [92]. The model-controlled image displays atrial and ventricular filling, as well as valve opening and closing during the cardiac cycle. Students can pause the simulation and track the names of the cardiac cycle phases, atrial and ventricular pressures and volumes, pulmonary artery and aortic pressures, and the current point on the pressure-volume loop. The multimedia tutorial on the Web page [80] describes how to create this simulator from the original Modelica model. The simulator was mainly developed for the purpose of the tutorial. If it was intended for education, more plots and sliders would be required.

## Discussion

### Principal Results

We created the Bodylight.js toolchain to facilitate the development of interactive simulators based on Modelica models. In this report, we focus on describing an important new component of the toolchain (Bodylight.js Composer), which enables the creation of browser-based simulators. More information about the toolchain and its use is available on the Web page [80].

The goals for the toolchain were addressed. Importantly, both Bodylight.js Composer and the simulators it produces are *browser based* and *client side*. The system runs in the browser without the need of any server-side back-end. It is also possible to distribute a standalone platform-independent HTML file and run it in the browser without an internet connection. This is enabled by the use of Web technologies such as JavaScript, WebAssembly, and HTML. We believe it will be *future-proof* (unlikely to become obsolete), as it is mainly based on open-standard technologies accepted and implemented by every major software vendor. If any of the applications in the toolchain are discontinued, it should be easy to replace them with another tool. We also find it to be *user friendly*. The models are implemented in the *equation-based Modelica* language using a modeling environment of the modeler’s choice, for example, OpenModelica or Dymola. Animations are created in a professional industry standard tool, Adobe Animate. The Composer uses the drag-and-drop technique to visually compose the simulator, and it is distributed under the General Public License 3.0 *open source* license and is *freely available*. Therefore, it is available for anyone to use and implement within their open-source projects. To our knowledge, no other tool exists, which meets all our requirements.

This approach brings together the domains of modeling, Web technologies, and graphical design, which supports better interdisciplinary cooperation of teachers, modelers, software developers, and graphic designers.

The Bodylight.js Composer is a self-contained client-side application, and anything submitted does not leave the user’s device; thus, there are no particular security or privacy issues.

Several simulators were created in Bodylight.js, and it proved to be a convenient solution fulfilling the needs of the designers. It would be extremely time consuming to implement these applications without this versatile toolchain. The new Nephron, Blood Circulation, and Pressure-Volume Loop simulators were presented in this paper to demonstrate the capabilities of Bodylight.js. The Nephron simulator was recently used in didactic classes of pathological physiology for medical students and was very well received by both the students and the teacher.

### Comparison With Previous Work

The client-side approach has several advantages over the client-server solutions available today. First, the simulator is not bound by the computational limitations of the server. The scaling problem is bypassed by avoiding the necessity of any server-side computations. Instead, we simply serve a Web page, which can be hosted on any Web server. Second, the client-side solution does not require a low-latency connection to the server. Thus, the client-side approach avoids the need for expensive Web hosting services.

E-learning and distance learning are becoming increasingly important in the world and particularly important in developing countries, where teachers are not easily accessible by many potential students [93]. Furthermore, because reliable internet connections may not be available for many people [94] and a round-trip latency is often high [95] in developing countries, the client-side solution could be more suitable here.

### Limitations

The OpenModelica export to FMU for Co-Simulation is currently limited to the Euler solver, without the option to include any of the other solvers available in the OpenModelica compiler. Euler is the simplest solver available, and its numerical performance is generally poor compared with more advanced solvers included in OpenModelica. Thus, the export from OpenModelica is currently not viable for many models. We are often forced to export the FMU from the proprietary Dymola, and this will continue at least until the issue with OpenModelica FMU export has been resolved.

The Adobe Animate, which is used for creation of the animations, is a commercial tool. JavaScript code describing the animations using the EaselJS library may be written by hand as an alternative.

In situations of a computationally complicated model or multiple plots or animations in the simulator, the performance drop becomes noticeable. The slower frame rate or model update rate does not make the simulator useless, but the user experience is reduced.

Bodylight.js relies on relatively new Web technologies; therefore, only browsers after late 2017 are able to run our simulators. However, because running older browsers is a security risk, most browser vendors have already switched to an automatic update system.

### Future

We will extend OpenModelica so that it can use advanced solvers in the FMU for Co-Simulation. We plan to optimize the model calculation and graphics rendering to achieve higher frame rates. The composer is modular in design; therefore, we are planning to add support for new libraries, such as different charting libraries or even other animation libraries, and external code contribution is welcomed.

### Conclusions

The new toolchain facilitates the production of teaching simulators not only in physiology but also in other fields where the behavior of systems can be described by mathematical equations, including biology, physics, chemistry, engineering, and economics. Furthermore, the technology is not limited to education or academia. Anyone with the ability to model their system, whatever it may be, can use Bodylight.js to visually explain it to any interested party.

New generations of electronic textbooks combining texts with images, animations, multimedia, and interactive model-driven simulators are emerging. These textbooks allow for experimentation with the simulation of the particular systems being taught, which contributes to a deeper understanding of the topics of interest.

We recommend that the teaching materials be developed as platform independent in-browser applications, which do not require installation and can operate without an internet connection. Bodylight.js fulfills all these requirements and is free and available for anyone to use, which can only help to increase its impact. We hope that this project will help people better understand a multitude of diverse systems.

## Appendix

Multimedia Appendix 1 Bodylight.js Composer Bouncing Ball tutorial video.

Multimedia Appendix 2 Nephron simulator.

## Abbreviations

ADH: antidiuretic hormone
e-learning: electronic learning
FMI: Functional Mock-up Interface
FMU: Functional Mock-up Unit
GUI: graphical user interface
HTML: HyperText Markup Language

## References

[1] Fischer Q, Sbissa Y, Nhan P, Adjedj J, Picard F, Mignon A, Varenne O (2018). Use of simulator-based teaching to improve medical students' knowledge and competencies: randomized controlled trial. J Med Internet Res.

[2] Pennaforte T, Moussa A, Loye N, Charlin B, Audétat MC (2016). Exploring a new simulation approach to improve clinical reasoning teaching and assessment: randomized trial protocol. JMIR Res Protoc.

[3] Kofránek J, Mateják M, Privitzer P (2010). KOFRLAB: Laboratory of Biocybernetics and Computer Aided Learning.

[4] Kofranek J, Matousek S, Rusz J, Stodulka P, Privitzer P, Matejak M, Tribula M (2011). The atlas of physiology and pathophysiology: web-based multimedia enabled interactive simulations. Comput Methods Programs Biomed.

[5] Burkhoff D, Dickstein ML Harvi.

[6] Leisman S, Burkhoff D (2017). Use of an iPad app to simulate pressure-volume loops and cardiovascular physiology. Adv Physiol Educ.

[7] Kurtz TW, DiCarlo SE, Pravenec M, Ježek F, Šilar J, Kofránek J, Morris Jr RC (2018). Testing computer models predicting human responses to a high-salt diet. Hypertension.

[8] Andrews PS, Polack FA, Sampson AT, Stepney S, Timmis J (2010). University of York.

[9] Jacob C, Hallgrimsson B, Coderre S, Jamniczky H LINDSAY Virtual Human.

[10] Jacob C, von Mammen S, Davison T, Sarraf-Shirazi A, Sarpe V, Esmaeili A, Phillips D, Yazdanbod I, Novakowski S, Steil S, Gingras C, Jamniczky HA, Hallgrimsson B, Wright B (2012). LINDSAY virtual human: multi-scale, agent-based, and interactive. Adv Intell Model Simul.

[11] Wilensky U, Rand W (2015). An Introduction to Agent-Based Modeling: Modeling Natural, Social, and Engineered Complex Systems With NetLogo.

[12] Marshall BD, Galea S (2015). Formalizing the role of agent-based modeling in causal inference and epidemiology. Am J Epidemiol.

[13] Fachada N, Lopes V, Rosa A (2007). Agent-Based Modelling and Simulation of the Immune System: A Review. Proceedings of the 13th Portugese Conference on Artificial Intelligence.

[14] Zhang L, Athale CA, Deisboeck TS (2007). Development of a three-dimensional multiscale agent-based tumor model: simulating gene-protein interaction profiles, cell phenotypes and multicellular patterns in brain cancer. J Theor Biol.

[15] Azimi M, Bulat E, Weis K, Mofrad MR (2014). An agent-based model for mRNA export through the nuclear pore complex. Mol Biol Cell.

[16] van Dyke PH, Savit R, Riolo RL (1998). Agent-Based Modeling vs Equation-Based Modeling: A Case Study and Users’ Guide. Proceedings of the First International Workshop on Multi-Agent Systems and Agent-Based Simulation.

[17] Pulse: Physiology Engine.

[18] Bray A, Webb JB, Enquobahrie A, Vicory J, Heneghan J, Hubal R, TerMaath S, Asare P, Clipp RB (2019). Pulse physiology engine: an open-source software platform for computational modeling of human medical simulation. SN Compr Clin Med.

[19] Kofránek J, Kulhánek T, Mateják M, Ježek F, Šilar J (2017). Integrative Physiology in Modelica. Proceedings of the 12th International Modelica Conference.

[20] Kofránek J, Vu LD, Snáselová H, Kerekes R, Velan T (2001). GOLEM--multimedia simulator for medical education. Stud Health Technol Inform.

[21] Lipovszki G, Aradi P (2006). Simulating complex systems and processes in LabVIEW. J Math Sci.

[22] Kiel JW, Shepherd AP (1988). A graphic computer language for physiology simulations. Comput Life Sci Educ.

[23] Lin SL, Guo NR, Chiu CC (2012). Modeling and simulation of respiratory control with LabVIEW. J Med Biol Eng.

[24] Cole RT, Lucas CL, Cascio WE, Johnson TA (2005). A LabVIEW model incorporating an open-loop arterial impedance and a closed-loop circulatory system. Ann Biomed Eng.

[25] Life Science Teaching Resources Community.

[26] Samosky JT, Nelson DA, Wang B, Bregman R, Hosmer A, Mikulis B, Weaver R (2012). BodyExplorerAR: Enhancing a Mannequin Medical Simulator With Sensing and Projective Augmented Reality for Exploring Dynamic Anatomy and Physiology. Proceedings of the Sixth International Conference on Tangible, Embedded and Embodied Interaction.

[27] Kofránek J, Mateják M, Privitzer P, Tribula M (2008). Laboratory of Biocybernetics and Computer Aided Learning.

[28] (2018). National Instruments.

[29] Jerome J (2010). Virtual Instrumentation Using LabVIEW.

[30] (2018). National Instruments.

[31] National Instruments.

[32] WebVIs: Developing Your Web-Based User Interface.

[33] Just Physiology.

[34] Meyer zu Eissen S, Stein B (2006). Realization of web-based simulation services. Comput Ind.

[35] Tiller MM, Winkler D (2017). modelica.university: A Platform for Interactive Modelica Content. Proceedings of the 12th International Modelica Conference.

[36] Tiller MM (2019). Modelica University.

[37] Žáková K, Cech M (2018). Design of Control Education Interactive Examples via Web Service for OpenModelica. Proceedings of the 13th APCA International Conference on Automatic Control and Soft Computing.

[38] JSON-RPC Working Group (2004). JSON-RPC.

[39] Raaen K, Grønli TM (2014). Latency Thresholds for Usability in Games: A Survey. Proceedings of the Norwegian Informatics Conference.

[40] McManus JP, Day TG, Mailloux ZJ (2019). Digital WPI: Worcester Polytechnic Institute.

[41] Wagner G (2017). Sim4edu.com: Web-Based Simulation for Education. Proceedings of the Winter Simulation Conference.

[42] Silar J, Kofranek J (2011). Physiological model creation and sharing. Eur J Biomed Inform.

[43] Short T (2019). GitHub Inc.

[44] BabylonJS.

[45] Batista AV, Lemos RR, Rudolph CM, Bueno BS, Fiuza PJ, Conceicao KR, Pereira PF, Mansur SS (2017). Design of A Web3D Serious Game for Human Anatomy Education. Handbook of Research on Immersive Digital Games in Educational Environments.

[46] Blender.

[47] BabylonJS Documentation.

[48] ThreeJS.

[49] Kasinathan V, Mustapha A, Nur FA, Zainal AA (2018). Three-dimensional e-learning application for anatomy and physiology of brain. Int J Integr Eng.

[50] Unity.

[51] Trivellato M (2018). Unity.

[52] Horachek D (2014). Creating eLearning Games With Unity.

[53] Zarzuela MM, Pernas FJ, Martínez LB, Ortega DG, Rodríguez MA (2013). Mobile serious game using augmented reality for supporting children's learning about animals. Procedia Comput Sci.

[54] Coelho A, Kato E, Xavier J, Gonçalves R (2011). Serious Game for Introductory Programming. Proceedings of the Second International Conference on Serious Games Development and Applications.

[55] George AK, McLain ML, Bijlani K, Jayakrishnan R, Bhavani RR (2016). A Novel Approach for Training Crane Operators: Serious Game on Crane Simulator. Proceedings of the Eighth International Conference on Technology for Education.

[56] Boada I, Rodriguez-Benitez A, Garcia-Gonzalez JM, Olivet J, Carreras V, Sbert M (2015). Using a serious game to complement CPR instruction in a nurse faculty. Comput Methods Programs Biomed.

[57] Gaudina M, Zappi V, Bellanti E, Vercelli G (2013). eLaparo4D: A Step Towards a Physical Training Space for Virtual Video Laparoscopic Surgery. Proceedings of the Seventh International Conference on Complex, Intelligent, and Software Intensive Systems.

[58] Jezek F, Tribula M, Kulhanek T, Matejak M, Privitzer P, Silar J, Kofranek J, Lhotska L (2015). Surviving Sepsis - a 3D Integrative Educational Simulator Internet. Proceedings of the 37th Annual International Conference of the IEEE Engineering in Medicine and Biology Society.

[59] Mattsson SE, Elmqvist H, Otter M (1998). Physical system modeling with Modelica. Control Engineering Practice.

[60] Tiller M (2012). Introduction to Physical Modeling with Modelica Internet.

[61] Fritzson P (2015). Principles of Object-Oriented Modeling and Simulation with Modelica 3.3: A Cyber-Physical Approach.

[62] Zimmer D (2016). Equation-based modeling with Modelica – principles and future challenges. Simul Notes Eur.

[63] Fritzson P, Engelson V (1998). Modelica - A Unified Object-Oriented Language for System Modeling and Simulation Internet. Proceedings of the 12th European Conference on Object-Oriented Programming.

[64] Mateják M, Kulhánek T, Šilar J, Privitzer P, Ježek F, Kofránek J (2014). Physiolibrary - Modelica Library for Physiology. Proceedings of the 10th International Modelica Conference.

[65] Mateják M Physiolibrary.

[66] OpenModelica.

[67] Dassault Systèmes.

[68] Kofránek J, Ježek F, Mateják M (2018). Modelica Language - A Promising Tool for Publishing and Sharing Biomedical Models. Proceedings of the 1st American Modelica Conference.

[69] Blochwitz T, Otter M, Akesson J, Arnold M, Clauss C, Elmqvist H, Friedrich M, Junghanns A, Mauss J, Neumerkel D, Olsson H, Viel A (2012). Functional Mockup Interface 2.0: The Standard for Tool Independent Exchange of Simulation Models. Proceedings of the 9th International Modelica Conference.

[70] (2014). Modelica.

[71] Flanagan D (2006). JavaScript: The Definitive Guide.

[72] W3Schools.

[73] Arseniev A GrapesJS.

[74] Skinner G CreateJS.

[75] Plotly: Graphing Libraries.

[76] Zakai A WebAssembly.

[77] Zakai A Emscripten.

[78] Zakai A (2011). Emscripten: An LLVM-to-JavaScript Compiler. Proceedings of the ACM International Conference Companion on Object Oriented Programming Systems Languages and Applications Companion.

[79] Falliere N (2018). PNF Software.

[80] Polak D, Šilar J, Ježek F, Mladek A, Kofranek J Bodylight.js.

[81] Dassault Systèmes.

[82] Polák D GitHub.

[83] Polák D GitHub.

[84] Adobe.

[85] Polák D, Šilar J BodylightJS.

[86] Polák D, Šilar J, Kofránek J BodylightJS.

[87] Šilar J, Mladek A, David A, Živny J, Kofranek J http://physiome.cz/apps/Nephron/.

[88] Šilar J, Ježek F, Mládek A, Polák D, Kofránek J (2019). Model Visualization for e-Learning, Kidney Simulator for Medical Students. Proceedings of the 13th International Modelica Conference.

[89] David P Laboratory of Biocybernetics and Computer Learning Support.

[90] Kofranek J, David P Laboratory of Biocybernetics and Computer Learning Support.

[91] Ježek F, Kulhánek T, Kalecký K, Kofránek J (2017). Lumped models of the cardiovascular system of various complexity. Biocybern Biomed Eng.

[92] Burkhoff D, Tyberg JV (1993). Why does pulmonary venous pressure rise after onset of LV dysfunction: a theoretical analysis. Am J Physiol.

[93] Sife AS, Lwoga ET, Sanga C (2007). New technologies for teaching and learning: challenges for higher learning institutions in developing countries. Int J Educ Dev.

[94] Geissbuhler A, Bagayoko CO, Ly O (2007). The RAFT network: 5 years of distance continuing medical education and tele-consultations over the internet in French-speaking Africa. Int J Med Inform.

[95] Chavula J, Feamster N, Bagula A, Suleman H, Nungu A, Pherson B, Sansa-Otim J (2015). Quantifying the effects of circuitous routes on the latency of intra-Africa internet traffic: a study of research and education networks. e-Infrastructure and e-Services for Developing Countries.

