# Supplementary material for: Development of In-Browser Simulators for Medical Education: Introduction of a Novel Software Toolchain
Source: J Med Internet Res. 2019 Jul 3;21(7):e14160. doi: 10.2196/14160 (PMC6786851; doi:10.2196/14160)
Supplement: Multimedia Appendix 2 [file jmir_v21i7e14160_app2.zip › Nephron-Static/0_index.html]

Nephron


## 1. Glomerulus

## 2. Proximal Tubule

## 3. Loop of Henle

## 4. Distal tubule and collecting tubulus

## 5. Nephron

### Ohm’s law in hydraulics

The water flow analogy for electricity is useful for explaining voltage, current, and power. In general terms, charge is water, voltage is the pressure of water, current is the flow of the water. Power is the total amount of water flowing in given time. It is possible to have a small pipe with high pressure, or a large pipe with low pressure each passing the same amount of water. Increasing the height of the water reservoir increases the potential energy of the water (voltage). Resistance can be explained as the roughness of the width of the pipe.

### Diffusion

Diffusion refers to the process by which molecules intermingle as a result of their kinetic energy of random motion. Consider two containers of gas A and B separated by a partition. The molecules of both gases are in constant motion and make numerous collisions with the partition. If the partition is removed, the gases will mix because of the random velocities of their molecules. In time a uniform mixture of A and B molecules will be produced in the container. The tendency toward diffusion is very strong even at room temperature because of the high molecular velocities associated with the thermal energy of the particles.

### Osmosis

If two solutions of different concentration are separated by a semipermeable membrane which is permeable to the smaller solvent molecules but not to the larger solute molecules, then the solvent will tend to diffuse across the membrane from the less concentrated to the more concentrated solution. This process is called osmosis. Osmosis is of great importance in biological processes where the solvent is water. The transport of water and other molecules across biological membranes is essential to many processes in living organisms. The energy which drives the process is usually discussed in terms of osmotic pressure.

### Active transport

To move substances against a concentration or electrochemical gradient, the cell must use energy. This energy is harvested from ATP generated through the cell’s metabolism. Active transport mechanisms, collectively called pumps, work against electrochemical gradients. Small substances constantly pass through plasma membranes. Active transport maintains concentrations of ions and other substances needed by living cells in the face of these passive movements. Much of a cell’s supply of metabolic energy may be spent maintaining these processes. Because active transport mechanisms depend on a cell’s metabolism for energy, they are sensitive to many metabolic poisons that interfere with the supply of ATP.

Two mechanisms exist for the transport of small-molecular-weight material and small molecules. Primary active transport moves ions across a membrane and creates a difference in charge across that membrane, which is directly dependent on ATP. Secondary active transport describes the movement of material that is due to the electrochemical gradient established by primary active transport, which does not directly require ATP.

#### Uniport

When a particular type of molecule moves across a membrane, through carrier protein, independent of other molecule, the diffusion is called uniport.

#### Symport

When two kinds of molecules move in the same direction while diffusing through carrier proteins, it is called symport.

#### Antiport

When two kinds of molecules move in the opposite directions while diffusing through carrier proteins, it is called antiport.
