# Supplementary material for: Development of In-Browser Simulators for Medical Education: Introduction of a Novel Software Toolchain
Source: J Med Internet Res. 2019 Jul 3;21(7):e14160. doi: 10.2196/14160 (PMC6786851; doi:10.2196/14160)
Supplement: Multimedia Appendix 2 [file jmir_v21i7e14160_app2.zip › Nephron-Static/1_glomerulus.html]

Blood enters the afferent arteriole (with hydrostatic pressure Pa and plasma osmolarity 300 mOsm/l), flows into the glomerulus, a cluster of intertwined capillaries (pressure Pg) and exits via the efferent arteriole (hydrostatic pressure Pe and plasma osmolarity 300 mOsm/l). The Bowman’s capsule (also called the glomerular capsule) surrounds the glomerulus and is composed of two layers: visceral and parietal.  
  
The visceral layer lies just beneath the thickened glomerular basement membrane and is made of podocytes that form small slits in which the fluid passes through into the nephron. The size of the filtration slits restricts the passage of large molecules (albumin) and cells (red blood cells, platelets, etc.) that are the non-filterable components of blood.  
  
These then leave the glomerulus through the efferent arteriole, which becomes capillaries meant for kidney–oxygen exchange and reabsorption before becoming venous circulation. The positively charged podocytes will impede the filtration of negatively charged particles as well.  
  
The process by which glomerular filtration occurs is called renal ultrafiltration (yellow dashed arrows). The force of hydrostatic pressure in the glomerular vessels Pg (the force of pressure exerted from the pressure of the blood vessel itself) is the driving force that pushes filtrate out of the capillaries and into the slits in the nephron. Oncotic pressure πg (~ 30 mmHg), the pulling force exerted by the albumins, works against the greater force of hydrostatic pressure, and the difference between the two determines the effective pressure of the glomerulus (Pg - πg), i.e. the total force that pushes molecules out of the capillaries. However, to get the real force by which molecules are filtered we need to consider also the hydrostatic (Pb ~ 10 mmHg) and the oncotic (πb ~ 0 mmHg as there are virtually no proteins) pressure inside the glomerulus (yellow column). To sum it up, the net glomerular filtration rate (GFR) is proportional to:

where Kf is the constant of proportionality which takes capillary permeability and other factors into account. Pressures are depicted with the liquid-column gauge, flows through the tubules with the pointer measures and turbines, flow through the vessel walls with width and speed of the dashed moving arrows. This visualised sensors are used also in all other parts of the application.

A student can change resistances of afferent and efferent arterioles and thus effectively control the hydrostatic pressure in glomerular capillaries and therefore GFR. The mean arterial pressure (MAP), which affects the pressure at the afferent arteriole entry may be also modified. The goal of the current slide is to explain how the glomerulus maintains approximately constant GFR despite changing arterial pressure by means of changing afferent and efferent arteriole resistances.
