# Supplementary material for: Development of In-Browser Simulators for Medical Education: Introduction of a Novel Software Toolchain
Source: J Med Internet Res. 2019 Jul 3;21(7):e14160. doi: 10.2196/14160 (PMC6786851; doi:10.2196/14160)
Supplement: Multimedia Appendix 2 [file jmir_v21i7e14160_app2.zip › Nephron-Static/2_proximal_tubule.html]

The proximal tubule is the major resorptive segment of the nephron and accounts for resorption of nearly two-thirds of all filtered water, sodium, and chloride. In addition, the proximal tubule is the segment where the majority of critical organic solutes such as glucose and amino acids are resorbed. Finally, this segment also plays an important role in acid-base balance as it is involved in bicarbonate reabsorption and secretion of organic acids.

The number inside the tubule depicts the osmolarity of the filtrate. Decrease of flow may be observed on the flow measures whereas the osmolarity is maintained throughout whole proximal tubulus. Note that the flow of decreases along the tubulus due to the resorption of various molecules dragging solvent outside the tubulus. Using the slider student can change the glomerular filtration rate.
