# Supplementary material for: Development of In-Browser Simulators for Medical Education: Introduction of a Novel Software Toolchain
Source: J Med Internet Res. 2019 Jul 3;21(7):e14160. doi: 10.2196/14160 (PMC6786851; doi:10.2196/14160)
Supplement: Multimedia Appendix 2 [file jmir_v21i7e14160_app2.zip › Nephron-Static/3_henle.html]

The thin descending Loop of Henle displays a high permeability to water and solutes and is responsible for approximately 20% of water resorption, although transport of these molecules occurs completely passively following the osmolarity gradient. The respective flow and osmolarity plot is shown in green.

The ascending loop of Henle accounts for resorption of nearly a quarter of the filtered load of sodium, chloride, and potassium ions. In addition, Henle's thick segment is a major location of magnesium and calcium ion resorption. Importantly, the tight junctions of this segment are virtually impermeable to water. Given the large amount of solute resorption that occurs in the absence of water resorption, the tubular fluid becomes progressively dilute as it travels through the thick ascending loop. This feature is why this segment is frequently referred to as the "Diluting Segment" of the nephron. The respective flow and osmolarity plot is shown in pink.

  

Try to change glomerular filtration rate and note the adjustment of flow and osmolarity. Clicking on “reset simulation” button will set GFR to the default value. The actual flow is visualized by the propeller rotation velocity. Pay attention also to the peak osmolarity value, 1200 mOsm/l corresponding to the osmolarity of the deep medulla areas.
