# Supplementary material for: Development of In-Browser Simulators for Medical Education: Introduction of a Novel Software Toolchain
Source: J Med Internet Res. 2019 Jul 3;21(7):e14160. doi: 10.2196/14160 (PMC6786851; doi:10.2196/14160)
Supplement: Multimedia Appendix 2 [file jmir_v21i7e14160_app2.zip › Nephron-Static/4_dt_ct.html]

The early distal tubule of the nephron is responsible for resorption of approximately 5% of the filtered load of sodium and chloride. Like the ascending loop of Henle, the early distal tubule is relatively impermeable to water and thus the tubular fluid becomes even further diluted as it travels through this segment. Additionally, the distal tubule is also responsible for 8% of the filtered calcium load and is the key segment of regulated calcium resorption.  
The late distal tubule and collecting ducts represent the final functional segment of the nephron after which any remaining tubular fluid is excreted as urine. By this segment, the vast majority of solutes and water have been resorbed and thus the late distal tubule and collecting ducts are only responsible for a small fraction of total resorption. However, this represents the major locus of regulated tubular resorption and given the enormous quantities of glomerular filtration that occur per minute, even small changes in resorption rates at this segment can have enormous impacts on the composition of the body's extracellular fluid. Modulation of water, sodium, chloride, and potassium resorption all occur in the late distal tubule and collecting duct. In addition, this segment is a major contributor to hydrogen excretion and thus plays an important role in acid-base homeostasis.

The terminal portion of the distal tubule empties through collecting tubules into a straight collecting duct in the medullary ray. The collecting duct system is under the control of antidiuretic hormone (ADH). When ADH is present, the collecting duct becomes permeable to water. The high osmotic pressure in the medulla (generated by the countercurrent multiplier system/loop of Henle) then draws out water from the renal tubule, back to vasa recta (not shown here).

The amount of ADH may be controlled by the student. Higher permeability of the tubule wall for water is represented by widening of the blue water channels. The goal is to explain how ADH affects the reabsorption: With no ADH, there is no reabsorption. Filtrate goes through unaffected. Urine production rate is high and its osmolarity is low. With full ADH so much water is reabsorbed that the urine osmolarity equalizes with osmolarity of surrounding the medulla. Only small amount of highly osmotic urine is produced.

Clicking on “reset simulation” button will set ADH and GFR to the default values. The osmolarity within distal tubule and collecting duct is plotted in purple and gold, respectively.
