# Supplementary material for: Development of In-Browser Simulators for Medical Education: Introduction of a Novel Software Toolchain
Source: J Med Internet Res. 2019 Jul 3;21(7):e14160. doi: 10.2196/14160 (PMC6786851; doi:10.2196/14160)
Supplement: Multimedia Appendix 2 [file jmir_v21i7e14160_app2.zip › Nephron-Static/5_nephron.html]

All the information from previous screens is recapitulated here. For simplicity the GFR is controlled directly instead of controlling afferent and efferent arteriole resistance as it was in the glomerulus section. ADH can be controlled as well. 

Filtrate flows at input and output are displayed both per nephron and per pair of kidneys. The Q indicators display the actual renal flow in l/day, QNa shows the sodium loss in g/day. Please note that Q significantly decreases from ~ 180 l/day, corresponding to GFR, down to less than 1 l/day.

The separate nephron parts are highlighted by different colors: proximal tubulus (orange), descending loop of Henle (green), ascending loop of Henle (pink), distal tubulus (purple), and the collecting duct (gold). The propeller rotation velocity indicates the flow inside the nephron. Please try various scenarios by setting GFR and ADH to respective values and explain what is going on.
