# Supplementary material for: Development of In-Browser Simulators for Medical Education: Introduction of a Novel Software Toolchain
Source: J Med Internet Res. 2019 Jul 3;21(7):e14160. doi: 10.2196/14160 (PMC6786851; doi:10.2196/14160)
Supplement: Multimedia Appendix 2 [file jmir_v21i7e14160_app2.zip › Nephron-Static/index.html]

Nephron


# Laboratory of biocybernetics and computer aided learning

<

>
