# Supplementary figures and images for: Development of In-Browser Simulators for Medical Education: Introduction of a Novel Software Toolchain
Source: J Med Internet Res. 2019 Jul 3;21(7):e14160. doi: 10.2196/14160 (PMC6786851; doi:10.2196/14160)

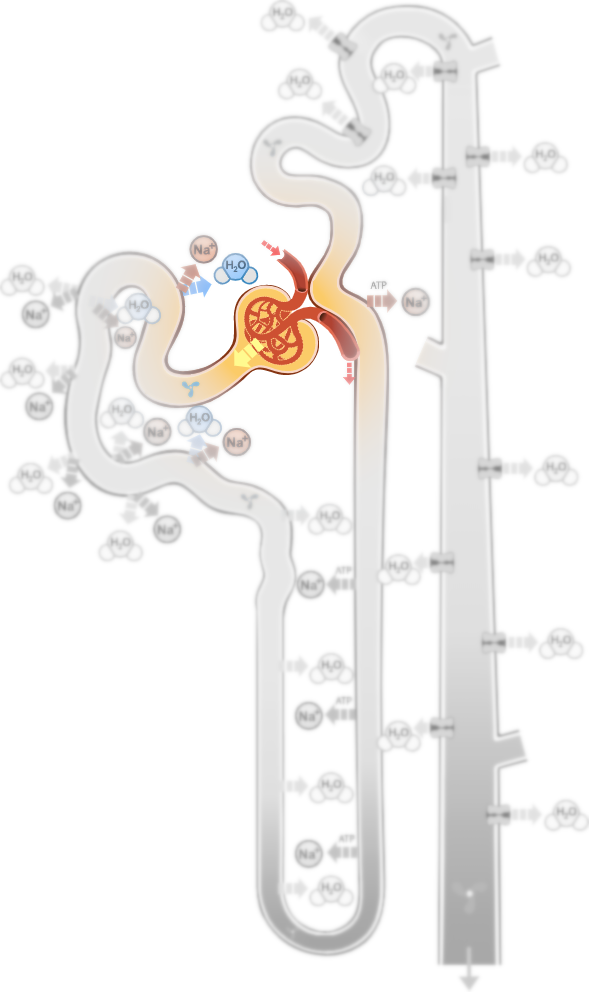

Supplement: Multimedia Appendix 2 [file jmir_v21i7e14160_app2.zip › Nephron-Static/img/1_glomerulus.png]

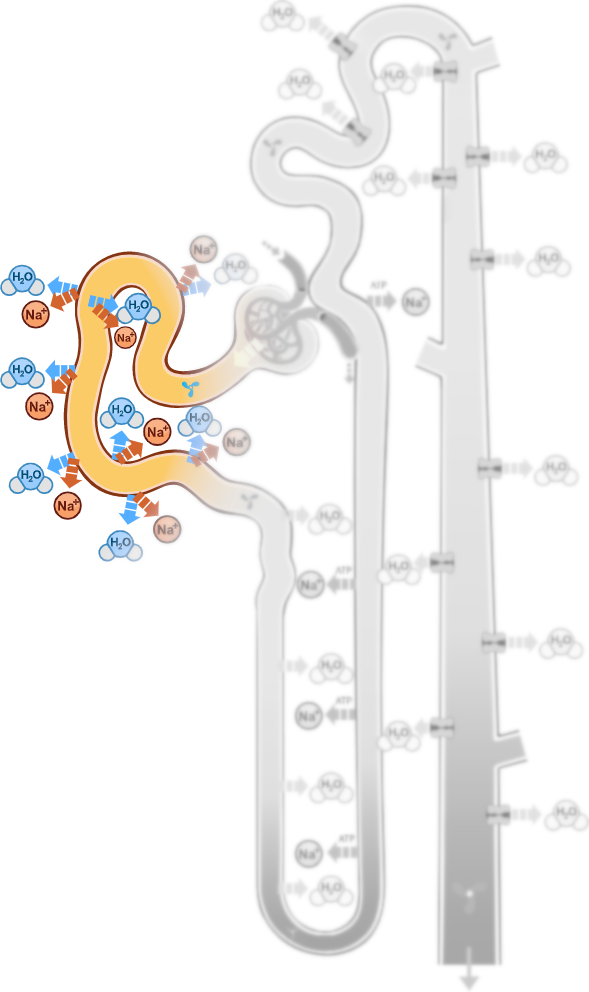

Supplement: Multimedia Appendix 2 [file jmir_v21i7e14160_app2.zip › Nephron-Static/img/2_proximal_tubule.png]

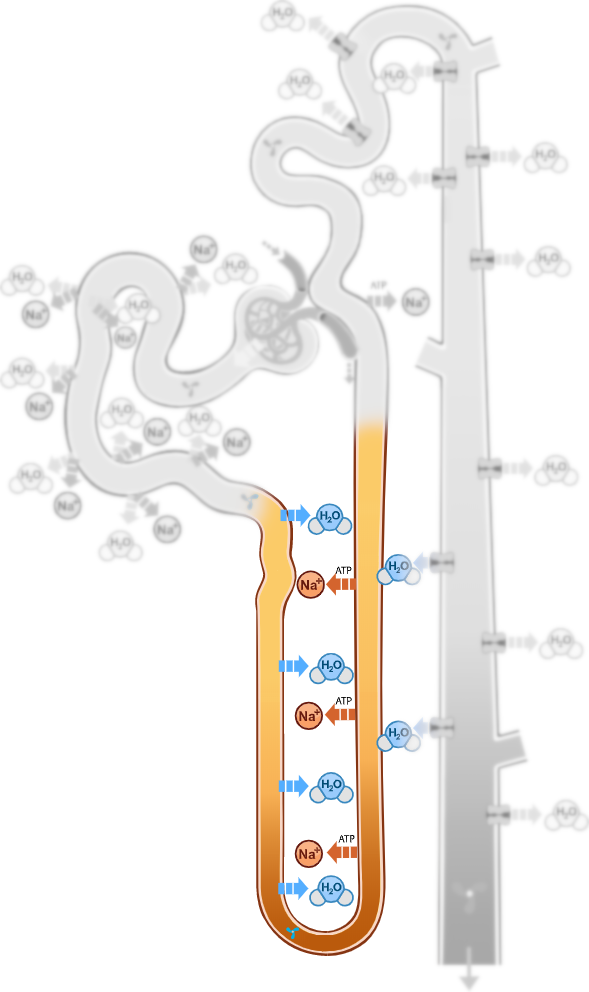

Supplement: Multimedia Appendix 2 [file jmir_v21i7e14160_app2.zip › Nephron-Static/img/3_henle.png]

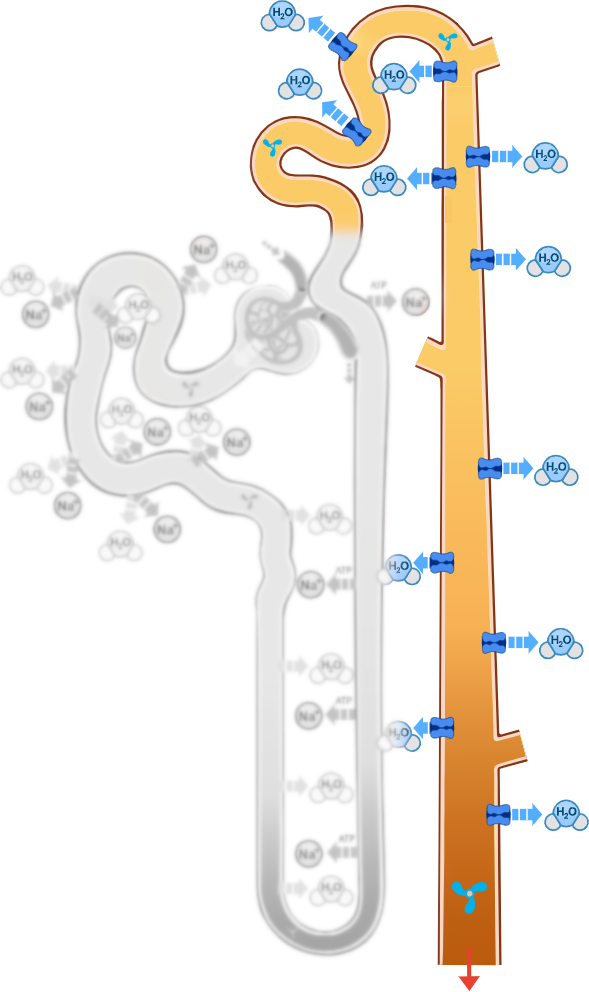

Supplement: Multimedia Appendix 2 [file jmir_v21i7e14160_app2.zip › Nephron-Static/img/4_dt_ct.png]

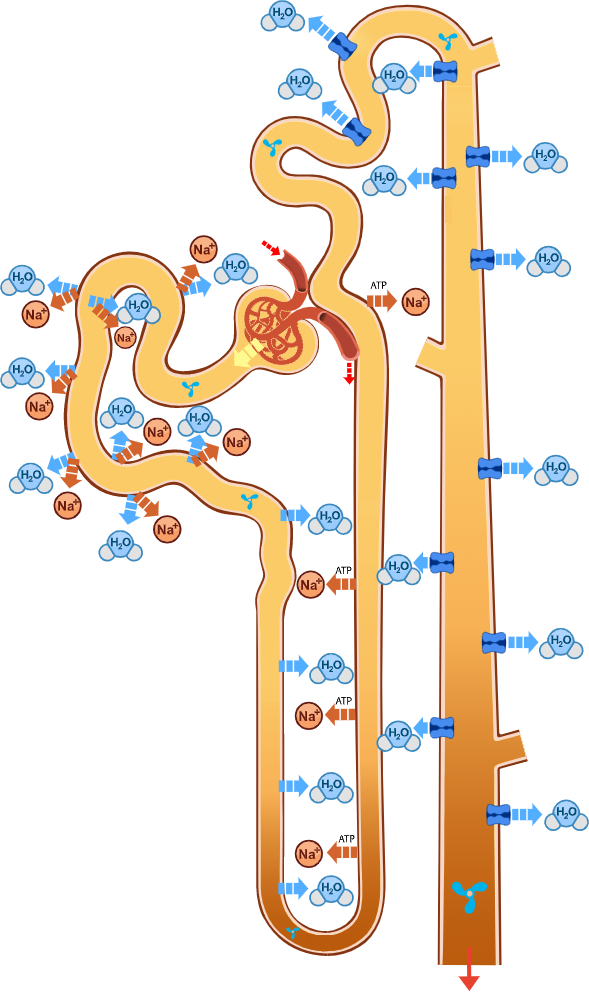

Supplement: Multimedia Appendix 2 [file jmir_v21i7e14160_app2.zip › Nephron-Static/img/5_nephron.png]

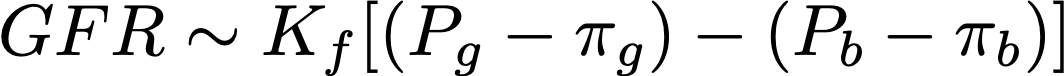

Supplement: Multimedia Appendix 2 [file jmir_v21i7e14160_app2.zip › Nephron-Static/img/gfr.png]

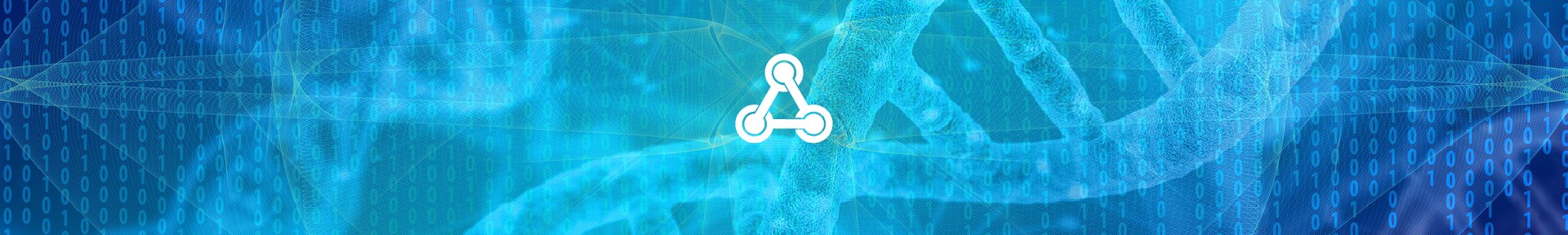

Supplement: Multimedia Appendix 2 [file jmir_v21i7e14160_app2.zip › Nephron-Static/img/header.jpg]

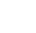

Supplement: Multimedia Appendix 2 [file jmir_v21i7e14160_app2.zip › Nephron-Static/img/logo-cc.png]

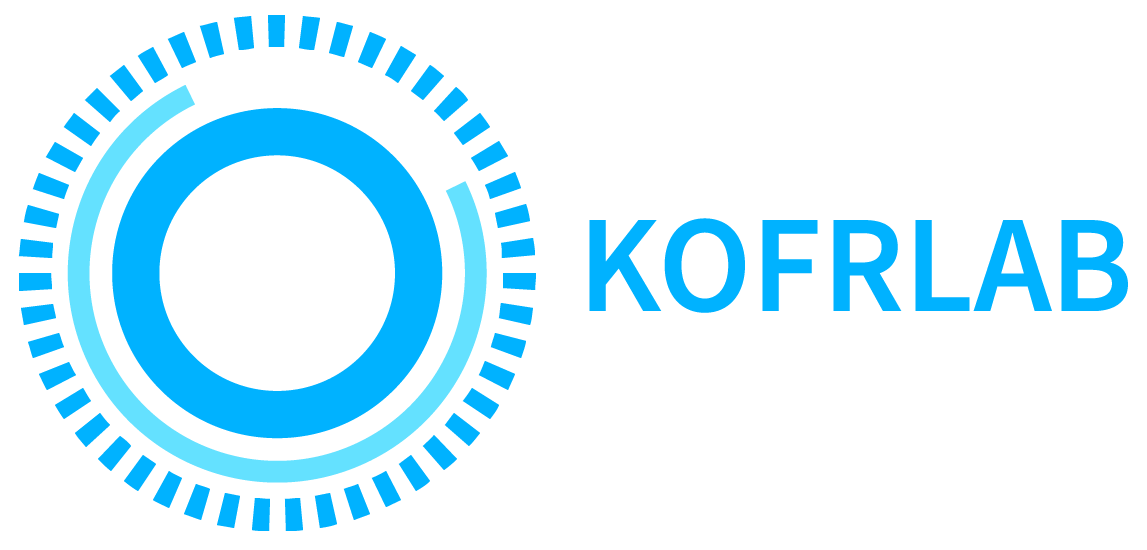

Supplement: Multimedia Appendix 2 [file jmir_v21i7e14160_app2.zip › Nephron-Static/img/logo.png]
